# Mapping Host-Related Correlates of Influenza Vaccine-Induced Immune Response: An Umbrella Review of the Available Systematic Reviews and Meta-Analyses

**DOI:** 10.3390/vaccines7040215

**Published:** 2019-12-13

**Authors:** Alexander Domnich, Ilaria Manini, Giovanna Elisa Calabrò, Chiara de Waure, Emanuele Montomoli

**Affiliations:** 1 Department of Molecular and Developmental Medicine, University of Siena, Via Aldo Moro 2, 53100 Siena, Italy; ilaria.manini@unisi.it (I.M.); emanuele.montomoli@unisi.it (E.M.); 2 Section of Hygiene, Institute of Public Health, Università Cattolica del Sacro Cuore, Largo Francesco Vito 1, 00168 Rome, Italy; alisacalabro@icloud.com; 3 Department of Experimental Medicine, University of Perugia, Piazza Lucio Severi 1, 06132 Perugia, Italy; chiara.dewaure@unipg.it; 4 VisMederi Life Science research, Strada del Petriccio e Belriguardo 35, 53100 Siena, Italy

**Keywords:** influenza, vaccines, vaccination, influenza vaccines, immunogenicity, correlates of protection, hemagglutination inhibition, umbrella review

## Abstract

Seasonal influenza is the leading infectious disease in terms of its health and socioeconomic impact. Annual immunization is the most efficient way to reduce this burden. Several correlates of influenza vaccine-induced protection are commonly used, owing to their ready availability and cheapness. Influenza vaccine-induced immunogenicity is a function of host-, virus- and vaccine-related factors. Host-related factors constitute the most heterogeneous group. The objective of this study was to analyze the available systematic evidence on the host factors able to modify influenza vaccine-induced immunogenicity. An umbrella review approach was undertaken. A total of 28 systematic reviews/meta-analyses were analyzed—these covered the following domains: intravenous drug use, psychological stress, acute and chronic physical exercise, genetic polymorphisms, use of pre-/pro-/symbiotics, previous Bacillus Calmette–Guérin vaccination, diabetes mellitus, vitamin D supplementation/deficiency, latent cytomegalovirus infection and various forms of immunosuppression. In order to present effect sizes on the same scale, all possible meta-analyses were re-performed and cumulative evidence synthesis ranking was carried out. The meta-analysis was conducted separately on each health condition category and virus (sub)type. A total of 97 pooled estimates were used in order to construct an evidence-based stakeholder-friendly map. The principal public health implications are discussed.

## 1. Introduction

Influenza is the world’s leading annually occurring infectious disease and places an enormous burden on public health [1]. For instance, in the general European population, it ranks first in terms of both attack and mortality rates (surpassing, for example, tuberculosis and human immunodeficiency virus (HIV) infection), and results in an average annual loss of 81.8 disability-adjusted life years per 100,000 inhabitants [2].

Annual influenza vaccination is the main public health intervention able to reduce the burden of disease [1,3]. Most currently available influenza vaccines (IVs) are egg-derived, inactivated (either split or subunit), trivalent or quadrivalent [4,5]. The World Health Organization’s (WHO) most recent position paper [1] has recognized some priority target groups for annual influenza vaccination, including pregnant women, children aged 6 months to 5 years, the elderly, subjects with specific chronic conditions, healthcare workers and international travellers. Despite the recommendations of the WHO (which has also adopted the value-based approach) [1], seasonal influenza vaccine policies are well-established only in high- and a few middle-/low- income countries [6]. In most countries of the WHO European Region, for example, vaccination is recommended for the elderly, people with underlying risk conditions, institutionalized populations, healthcare workers and (in fewer countries) children and pregnant women [7], while in the United States (US), it is universally recommended (for all subjects aged 6 months and above) [8].

IV-induced protection is ideally measured by conducting randomized controlled trials (RCTs) in which the clinical endpoint is laboratory-confirmed influenza. Such studies are, however, expensive. Moreover, their execution is hindered by some particularities of influenza, including its variable annual attack rates, extremely high seasonality pattern in most countries, frequent viral mutations, heterogeneity of the circulating virus population, and varying annual vaccine composition [9]. This is why most IV RCTs use immunogenicity parameters as their primary endpoints. In a simplistic way, it may be claimed that IV-induced protection against influenza is entirely mediated by a single surrogate endpoint (i.e., immune response). In other words, the IV-induced protection must correlate with the IV-induced immune response [10]. Plotkin [11] has defined a correlate of vaccine-induced protection as “*an immune response that is responsible for and statistically interrelated with protection*”—in this paper, we will adopt this definition.

The hemagglutination-inhibition assay (HAI) is the most commonly used assay in IV RCTs. Historically, a HAI threshold titer of 1:40 was associated with a 50% reduction in the absolute risk of contracting influenza [12]. This figure comes from an adult challenge study dating back to the early ‘70s [13]. More recent meta-analytical models, however, seem to agree to some extent with that estimate. Specifically, De Jong et al. [14] estimated that the HAI titer of 1:192 was associated with a 90% median reduction in influenza. The Bayesian meta-analytical approach adopted by Coudeville et al. [15] established that the incremental increase in clinical protection was very marked at HAI titers of up to 1:100, while the benefit became marginal at titers >1:150. Nonetheless, the above-described HAI “universal” threshold of 1:40 may not be appropriate for some population groups. Indeed, it has been estimated [16] that, in children, the conventional HAI titer cut-off of 1:40 is associated only with a 22% protection rate, while the cut-offs of 1:110, 1:215, 1:330 and 1:629 predict 50%, 70%, 80% and 90% protection, respectively.

Other, less commonly used, immunological assays are single-radial hemolysis, virus neutralization and enzyme-linked immunosorbent (ELISA) assays [12]. Among these, only single-radial hemolysis has an established correlate of protection threshold, which is ≥25 mm^2^ lysis zone; this roughly corresponds to the HAI cut-off titer of 1:40 [17]. Apart from the humoral immune response correlates of protection, cellular ones are increasingly being recognized and investigated [18].

Schematically, the immunogenicity, efficacy and effectiveness of IVs may be deemed to be a function of the characteristics of the host, virus and vaccine [19]. Among the host factors, the age of the vaccinee is probably the factor most known to affect the immunogenicity of IVs. While a poor immune response in the youngest age-class is usually ascribed to the immaturity of the immune system [20], in the elderly it has been linked to immunosenescence [21]. The factors related to the influenza virus are well-known—intense selection by the host immune system drives antigenic change in both A and B virus types and results in the continuous replacement of circulating strains with new ones that can re-infect hosts that are immune to previously circulating variants (the phenomenon known as “antigenic drift”) [22]. In the past few years, however, the “vaccine-related” factor has received more attention, owing to the continuously diversifying IV market. For instance, adjuvanted, intradermal and high-dose IVs have consistently been shown to enhance immunogenicity in comparison with standard-dose intramuscularly administered non-adjuvanted vaccines [23].

Several intrinsic host characteristics (e.g., age, sex, genetic polymorphisms, and many morbidities) can be viewed as unmodifiable factors that may alter the immune response following vaccination. On the other hand, modifiable host-related factors (e.g., lifestyle habits or dietary patterns) may also potentially interfere with IV-induced immunogenicity. A down-to-earth appraisal of these factors and their impact on the IV-induced immune response is essential both to the design of future immunogenicity RCTs and to the potential development of better and/or more personalized IVs [24] or, at least, IV-related public health policies. A recently published narrative review [25] examined some host factors that affect the immune response to IV, and the following factors were discussed: preexisting immunity, immunosenescence, genetic polymorphism, sex, obesity and the presence of chronic underlying medical conditions. In the present paper, we adopted a comprehensive approach, in that we conducted an analysis of the evidence from the available systematic analyses of the intrinsic host factors affecting IV-induced immunogenicity.

## 2. Materials and Methods

### 2.1. Objective

The objective was to analyze and graphically synthetize the available systematic evidence on the host factors able to modify IV-induced immunogenicity.

### 2.2. General Methodology

The present paper was conceived as an umbrella review. The umbrella review (also known as the overview/review of (systematic) reviews, meta-review, and similar) is an emerging field of evidence-based medicine and is becoming increasingly common, given the growing number of systematic reviews on the same/similar topics. The key feature of umbrella reviews is that they are focused on the highest possible level of evidence, i.e., systematic reviews (SRs) and meta-analyses (MAs) or both (SRMAs). Furthermore, umbrella reviews are seen as a ready means of enabling relevant stakeholders to gain a clear understanding of a broad topic area [26]. Indeed, the host factors affecting IV-induced immunogenicity constitute a vast and heterogeneous group of modifiers.

This study forms part of a PhD thesis by A.D. No ethical approval was deemed necessary, given the second-hand nature of this research.

### 2.3. Search Strategy

The search strategy was first calibrated by using a simplistic search string implemented in Google Scholar (www.scholar.google.com): (“influenza” AND “vaccine” AND (“immunogenicity” OR “immune response”) AND (“systematic review” OR “meta analysis”)). We then examined the first 500 search results and selected potentially eligible papers. We selected only the first 500 results, given the low specificity of Google Scholar: the search produced more than 16,000 results. Google Scholar was chosen since, unlike well-established scientific databases, it can work well with the so-called “gray literature” [27]. A more detailed search strategy was then developed and tested on PubMed (www.ncbi.nlm.nih.gov/pubmed) in order to ensure that all records selected in Google Scholar appeared in the PubMed results output. The following PubMed script was then judged appropriate: ((((((“influenza” OR “flu”)) OR influenza, human[mh])) AND (((vaccin* OR immuni*)) OR vaccines[mh]))) AND ((review literature as topic[mh]) OR (“systematic review” OR “meta analysis” OR “meta regression”)). The above-described search algorithm was adapted to Embase (www.embase.com) and the search output was retrieved. We then searched the Cochrane Library (www.cochranelibrary.com); however, given that this focuses on SRs/SRMAs, the search was limited to the single keyword “influenza”, in order to increase its sensitivity. The automatic search outputs from the three databases were pooled in a single spreadsheet, and duplicates were removed in a semi-automatic modality. The last automatic search was performed on 2 July 2019 by A.D.

The automatic search was subsequently followed by a manual search. This included (i) standard cross-reference checking of the manuscripts included; (ii) checking articles that cited the SR/SRMAs included (through Google Scholar in order to check for possible sources of the “gray literature”); (iii) seeking advice on additional SR/SRMAs from academic experts/industry. We also tried to search the principal RCT registries (www.clinicaltrials.gov and www.clinicaltrialsregister.eu) and “gray literature” databases (www.opengrey.eu), though this proved fruitless.

We then updated (according to the last search time-period declared by the authors of the SRs/SRMAs included) the list of primary studies by applying the same search strategy and the same inclusion and exclusion criteria used in the SRs/SRMAs included.

### 2.4. Eligibility Criteria and Inclusion Process

All SRs or SRMAs concerning the host factors potentially affecting IV-induced immunogenicity were eligible. The inclusion criteria were formulated according to the PICO (population, intervention, control, outcome) framework. Specifically, no restrictions were placed on the population groups (e.g., age or health conditions) and settings. The intervention was IV of any type. Cases were defined as vaccinees with a given health condition that could modify the IV-induced immune response, while controls were vaccinees without that condition (usually healthy controls). The outcome of interest was the humoral immune response, as measured by HAI. This choice was based on our many years of experience of this topic (as witnessed by I.M. and E.M.). Moreover, HAI is (i) a relatively cheap, well-standardized assay with a well-established threshold as a correlate of protection [12] and (ii) is/was required by regulatory agencies in Europe [28] and the US [29].

The following statistical parameters associated with the HAI are commonly used and/or required: geometric mean titers (GMTs) following IV, seroconversion rate (SC), usually defined as proportion of vaccinees with at least a 4-fold increase from before to after IV, and seroprotection rate (SP), usually defined as the proportion of vaccinees with an HI titer ≥1:40 [28,29]. All these parameters were planned *a priori* for inclusion in the meta-synthesis.

SRs/SRMAs of both experimental and observational studies could be included in the analysis. RCTs are a well-known means of comparing two or more experimental arms in a relatively unbiased way, which is why the SRMAs of RCTs were our primary choice. However, several host factors that may potentially alter IV-induced immunogenicity are relatively rare in the general population; observational studies may, therefore, be more convenient than RCTs. Moreover, some ethical issues may arise from not offering IV to people for whom it is recommended. For this reason, we also decided to include SRs/SRMAs of observational studies (both cohort and case-control).

In the first step, we screened titles and/or abstracts of the combined duplicate-free search output for the following exclusion criteria: (i) animal or in vitro studies; (ii) no active immunization with IVs, (iii) no immunogenicity endpoints as correlates of protection (e.g., only efficacy, effectiveness, safety, acceptance and other irrelevant outcomes); (iv) non-systematic nature of the manuscript (e.g., narrative or expert-driven reviews), and (v) conference abstracts/proceedings with little available information. However, the reference lists of any identified narrative reviews on the topic of interest were screened.

All potentially eligible records and those whose eligibility was unclear from the title/abstract underwent full-text assessment. Full texts meeting all the inclusion criteria were included in the analysis unless they met the following exclusion criteria: (i) no predefined control group (e.g., the assessment of IV-induced immunogenicity in a given “ill” population, as in the case of cross-sectional study design); (ii) no separate information on IV-induced immunogenicity (i.e., an SR/SRMA dealing with vaccines against several diseases); (iii) control groups composed of unvaccinated individuals; (iv) SRs/SRMAs aimed at comparing different IV types; (v) MAs without a formal systematic search (in this case, however, the lists of primary studies included were assessed); (vi) SRs/SRMAs entirely focused on immunological assays other than HAI.

The study selection process was made by two reviewers (A.D. and I.M.), each working independently. Any disagreement was solved by discussion.

### 2.5. Data Extraction

Data were extracted and imported into an ad hoc spreadsheet by two reviewers (A.D. and I.M.), each working independently. Any disagreement was solved by discussion. The following data were extracted: first author and year of publication; review design (SR or SRMA); host factor(s) evaluated; study designs included (RCTs, observational or both); number of studies included (*k*); study population and setting; total number of cases and controls (see definitions above); statistical parameters of interest used to describe the immunogenicity endpoints; principal results of qualitative and quantitative syntheses; authors’ conclusions; funding sources; other potentially relevant information. When possible, the immunogenicity endpoints were extracted separately for each virus (sub)type (e.g., A/H1N1, A/H3N2, B).

### 2.6. Quality Appraisal

The SRs/SRMAs included were assessed by means of the AMSTAR-2 instrument (a measurement tool for assessing systematic reviews, version 2) [30]. This is an updated version of AMSTAR that is able to assess SRs of both RCTs and non-randomized studies; it consists of 16 items (instead of the 11 available in the previous version) and has simpler voting rules. The response categories are “Yes”, “Partial yes”, “No” and “0” (i.e., not applicable (NA), if, for example, no meta-analysis was performed). Of note, this instrument is not designed to produce a single overall SR/SRMA rating [30].

Once sufficiently trained by consulting the available comprehensive user guide [30], two reviewers (A.D. and G.E.C.) provided independent votes on each paper included. Any disagreements were solved by involving a third author (C.d.W.).

### 2.7. Data Analysis and Synthesis

Once extracted, the data from single manuscripts were first tabulated and summarized qualitatively. Results of SRs without MAs were summarized narratively and separately from SRMAs.

The papers included were then classified according to the host factor studied. If more than one SR/SRMA covered the same/similar host factor, we created citation matrices and quantified the corrected covered areas, as described by [31]. Specifically, the corrected covered area allows the overlap of primary studies included in different SRs to be quantified; it is expressed as (*N* − *r*)/(*rc* − *r*), where *N* is the number of papers included in all available SRs, *r* is the number of original primary studies, and *c* is the number of SRs. This overlap was categorized as slight (0%–5%), moderate (6%–10%), high (11%–15%), and very high (>15%) [31].

The results of single MAs were expressed in terms of different effect sizes, and the models adopted used different estimators. Moreover, some important information was sometimes missing from the meta-analytical output and/or was inadequately reported. In addition, we were able to identify some novel primary research studies. Consequently, we re-applied MAs by extracting the data from single primary studies (also considering the citation matrices described above) in order to be able to visualize the effect of different host factors on the same scale. We decided a priori to use both the random-effects and fixed-effects models. If the pooled random- and fixed-effects estimates differed substantially (as usually occurs in the case of high heterogeneity), the random-effects estimate was retained in our conclusions.

The pooled ESs for the binary outcomes of SC and SP were expressed as odds ratios (ORs) with corresponding 95% confidence intervals (CIs). For the continuous endpoint of post-immunization GMTs, Hedges’ *g* with 95% CIs was used. Given the skewed nature of GMTs, pooling was performed by means of log*_e_*-transformation, as recommended by the US Advisory Committee on Immunization Practices [32].

Heterogeneity was quantified by means of *I*^2^ and *τ*^2^. As per Cochrane’s Handbook [33], publication bias was assessed only for meta-analyses with *k* ≥ 10. Publication bias was assessed by computing Rücker’s arcsine and Egger’s tests for dichotomous and continuous endpoints, respectively. We decided not to test the excess significance bias formally, as proposed by Ioannidis and Trikalinos [34] since this test is not currently recommended by the Cochrane Collaboration [33]. Instead, we visually inspected the contour-enhanced funnel plots and noted substantial asymmetries. The 95% prediction intervals were also calculated in order to determine which range the next data point would probably lie.

Variability measures reported on different scales (e.g., 95% CIs, standard errors (SEs)) were converted to standard deviations (SDs), as recommended by the Cochrane Collaboration [33].

Missing values of the dispersion measures of GMTs were treated in a *post-hoc* modality. These values were inferred by averaging the available log*_e_*-transformed SDs from similar studies. We excluded studies in which the SDs had been inferred in sensitivity analyses in order to verify the robustness of the base case.

SC, SP rates and GMTs for the three virus (sub)types were pooled separately.

Having envisioned a sufficient number of meta-analyzed studies, we planned *a priori* to conduct a series of sensitivity analyses in order to determine the sources of the heterogeneity observed. For this purpose, we conducted subgroup and/or meta-regression analyses. The independent variables to include were selected *post hoc* on the basis of *k*, data availability and/or model fitting.

All statistical analyses were carried out by means of R (version 3.6.1) [35] and the stats packages “meta” [36] and “metafor” [37].

### 2.8. Cumulative Evidence Synthesis (CES)

Once all predefined summary effect sizes had been calculated, the following recommended rules [38] were applied in order to categorize the available evidence:

- Convincing (class I): pooled number of cases (*N*) > 1000, *p* < 0.000001, *I*^2^ < 50%, 95% prediction interval excluded the null, no small-study effect/publication bias;
- Highly suggestive (class II): *N* > 1000, *p* < 0.000001, largest study with a statistically significant effect (*p* < 0.05) and class I criteria not met;
- Suggestive (class III): *N* > 1000, *p* < 0.001 and class I–II criteria not met;
- Weak (class IV): *p* < 0.05 and class I–III criteria not met;
- Non-significant: *p* for the observed ES > 0.05.

As highlighted in the previous section, differently from the recommendations made by [38], we did not formally test the excess significance bias. However, the absence of excess significance bias is required only for CES class I. In the present paper, only 6/97 (6%) “mappable” estimates fell within CES I, and visual inspection of the contour-enhanced funnel plots did not suggest any excess of the “statistical significance” at *α* < 0.05.

Evidence mapping was then performed in order to contextualize the available evidence, identify gaps in the systematic research and present the data of this study in a readily intelligible way [39]. To do this, we created bubble plots considering the variables (i.e., CES and health condition) of interest.

## 3. Results

### 3.1. Selection Process and Main Characteristics of the Systematic Reviews and/or Meta-Analyses Included

**Figure 1:**
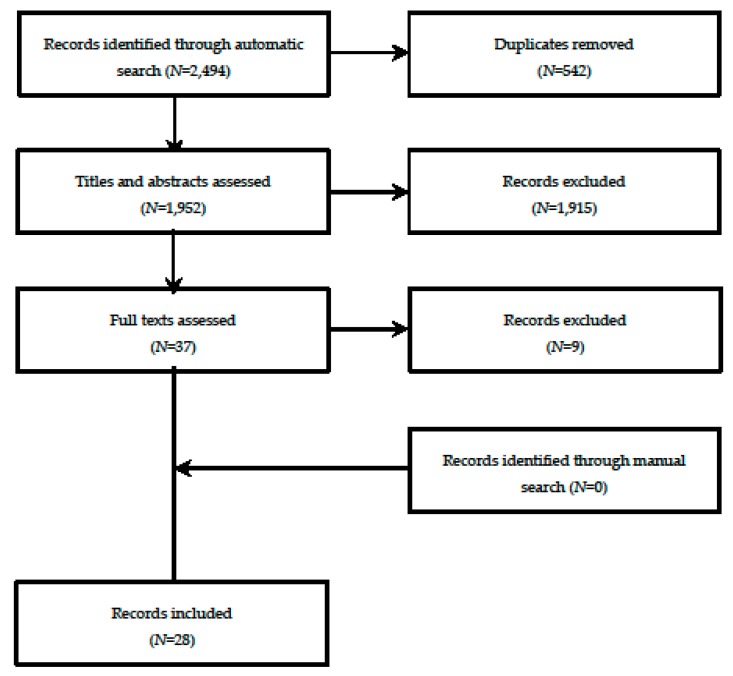
Record selection process.

The whole selection process is depicted in Figure 1. Briefly, the automatic search of three databases produced 2494 records, 542 of which were duplicates. The title/abstract screening procedure allowed us to eliminate further 1915 records as clearly ineligible. Of 37 full texts assessed, a total of 28 records [40,41,42,43,44,45,46,47,48,49,50,51,52,53,54,55,56,57,58,59,60,61,62,63,64,65,66,67] were retained in the present analysis. No eligible records were identified through manual search and expert/industry consultation. A list of the excluded studies, with reasons for exclusion, is provided in Supplementary Material (Table S1).

The main characteristics of the SRs/SRMAs included are reported in Table 1. Most (61%, 17/28) of the records included were SRMAs, while the remaining 39% were SRs without any quantitative synthesis. The following host factors were covered: intravenous drug use (*n* = 1), psychological stress (*n* = 1), acute exercise (i.e., a short bout of intensive physical activity) and chronic physical exercise (i.e., habitual physical activity, such as fitness training) (*n* = 1), genetic polymorphisms (*n* = 1), use of pre-/pro-/symbiotics (*n* = 3), BCG (Bacillus Calmette–Guérin) vaccination (*n* = 1), diabetes mellitus (*n* = 1), vitamin D supplementation/deficiency (*n* = 1), latent cytomegalovirus (CMV) infection (*n* = 1) and various forms of immunosuppression (*n* = 17). This last category included immunosuppressive conditions and/or drugs associated with rheumatic diseases, cancer, organ transplantation and inflammatory bowel disease, HIV, etc. (Table 1). However, we should point out that the classification adopted is a working one, since, for example, diabetes and intravenous drug use may also be seen as immunosuppressive conditions; we split these conditions into single categories on account of their particular public health burden.

**Figure 2:**
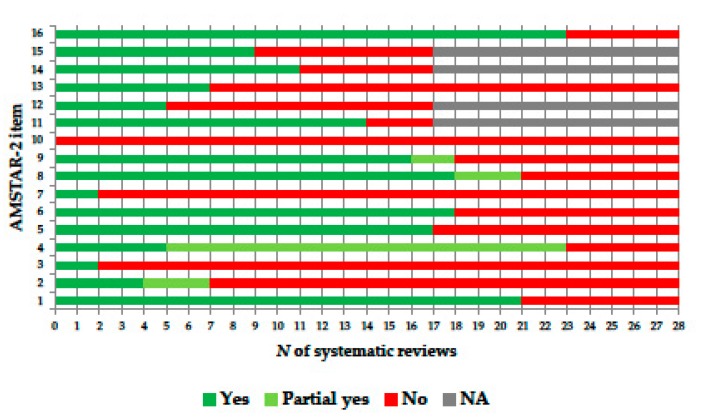
AMSTAR-2 (measurement tool for assessing systematic reviews, version 2) ratings, by item.

The number of the IV-related studies included in each SR/SRMA was highly skewed (range: 1–209) and presented a median of 15 (interquartile range: 9–18). With regard to the quality of reporting, no SR/SRMA met all 16 AMSTAR-2 criteria (Table 1). The most “problematic” AMSTAR-2 items were those regarding the explanation of the study designs for inclusion (item 3), an explicit list of the studies excluded (item 7) and consideration of the funding source of the primary studies included (item 10) (Figure 2). No correlation emerged between the number of “Yes” AMSTAR-2 votes and the year of SR publication (Spearman’s *ρ* = 0.07, *p* = 0.74). Detailed information on the AMSTAR-2 votes on the SRs included is provided in Supplementary Material, Table S2.

### 3.2. Use of Probiotics, Prebiotics or Symbiotics

Three SRs [58,64,65] evaluated the effect of using pro-/pre-/symbiotics on IV-induced immunogenicity. All three papers included only RCTs with at least two intervention arms, i.e., (i) intervention group: use of pro-/pre-/symbiotics and (ii) control group: placebo or other dietary supplements not containing pro-/pre-/symbiotics.

The SRMA by Lei et al. [58] pooled binary outcomes (SCs and SRs), while Yeh et al. [64] pooled post-vaccination HAI titers that were deemed to be “mean titers” throughout the results. Zimmermann et al. [65] did not conduct any MA. Some concerns regarding the pooling technique used by Yeh et al. [64] emerged. Specifically, (i) no transformation (e.g., log*_e_*) of the skewed summary antibody titers was undertaken, as shown by forest plots; (ii) an enormous variation in SDs; (iii) arithmetic and geometric means were pooled together.

A total of 23 meta-analytical estimates with at least two pooled primary RCTs were extracted from the two SRMAs [58,64]; the number of pooled studies ranged from 2 to 12, and more than half were statistically significant (*p* < 0.05) (Supplementary Material, Table S3). Briefly, Lei et al. [58] reported a statistically significant advantage of taking pro-/prebiotics in terms of immunogenicity with regard to all-age SC against type B and SP against A/H1N1 and A/H3N2. Yeh et al. [64] reported better results in the treatment arms in terms of the magnitude of the HAI titer against all three (sub)types; however, heterogeneity was extremely high (*I*^2^ = 94%–100%), casting doubt on the appropriateness of pooling single estimates. The narrative synthesis by Zimmermann et al. [65] concluded that the beneficial effect of probiotics on the IV-induced immune response was seen in 5 out of 12 (42%) studies analyzed. As expected (since all three SRs were published in a one-year period), the corrected covered area was very high (57.9%).

Given the above-described inconsistencies in effect size estimates, we re-pooled the available primary studies according to our methodology. Briefly, the total number of RCTs included ranged from 8 to 13. Statistically significant ORs were seen with regard to SP against type A viruses: subjects taking any pro-/pre-/symbiotic were significantly more likely to be protected against A/H1N1 and A/H3N2 (by 68% and 93%, respectively). With regard to SCs and post-vaccination HAI titers against all three (sub)types, there were no statistically significant differences between cases and controls. Table 2 reports the evidence synthesis of the effect of pro-/pre-/symbiotics on IV-induced immunogenicity. The level of evidence for the statistically significant estimates was, however, categorized as “class IV”.

We then conducted a subgroup analysis according to the type of supplement used (i.e., pro-/pre- or symbiotic) (Supplementary Material, Tables S4–S6). No definite conclusions could be drawn. Specifically, statistically significant ORs were seen in the following comparisons: (i) probiotic use and SC against A/H1N1 [2.89 (95% CI: 1.19, 6.99)] and (ii) prebiotic use and SP against A/H1N1 [2.72 (95% CI: 1.14, 6.50)]. Sub-analysis by age-class revealed two statistically significant comparisons: SC against A/H3N2 in working-age adults [OR 2.32 (95% CI: 1.07, 5.03)] and SP against A/H1N1 in the elderly [OR 2.40 (95% CI: 1.25, 4.60)]. The paucity of the available RCTs did not allow us to perform a meta-regression analysis.

On applying a post-hoc modality to each study included in the available SR/SRMAs, it emerged that most studies had been funded by dietary supplement producers, though this was not considered in the available SRMAs [58,64,65]. We, therefore, conclude that the estimates provided may be prone to the so-called “industry sponsorship” bias.

### 3.3. BCG (Bacillus Calmette–Guérin) Vaccination

One SR [66] investigated the effect of previous or concomitant BCG vaccination on the immunogenicity of various vaccines, including influenza. Three studies were included in the qualitative synthesis of this SR. The authors reported that these studies had found an enhanced effect of inactivated IVs on both the magnitude HAI antibodies and SC rates in recipients of a BCG vaccine. By contrast, no such effect was seen in subjects who received live attenuated IVs. It was not feasible to pool the results from the three studies, owing to the paucity of the available evidence, different study designs (randomized and non-randomized), different influenza vaccines (live, inactivated or both) and different timing of IV administration (concomitantly or separately from BCG).

### 3.4. Genetic Polymorphisms

An SR by Posteraro et al. [50] was the only one to investigate the potential role of genetic variations in the vaccine-induced immune response, including that induced by IVs. However, only one IV-related study was found and analyzed. Four statistically significant associations were reported. Positivity for HLA-DQB1*03:03 [OR 20.40 (95% CI: 1.10, 376.80)] and HLA-DRB1*07 [OR 4.00 (95% CI: 1.30, 12.20)] were associated with a negative IV-induced response, while positivity for HLA-DRB3*0X [OR 0.36 (95% CI: 0.10, 0.88)] and HLA-DRB1*13 [OR 0.18 (95% CI: 0.05, 0.69)] were associated with a positive response. No further studies were identified.

### 3.5. Intravenous Drug Use

Only one SR [40] evaluated the immunogenicity of different vaccines in intravenous drug users. Regarding influenza, only two primary studies were discussed. It is, however, difficult to draw any conclusion, as these two studies included former intravenous drug users who were all HIV-positive. HIV positivity as a host factor will be discussed below.

### 3.6. Vitamin D Supplementation/Deficiency

A recent SRMA by Lee et al. [62] investigated the effect of vitamin D deficiency on the IV-induced immune response. On pooling the binary outcomes of SCs and SPs, the authors obtained inconclusive results. Specifically, vaccinated subjects with normal vitamin D serum levels had a lower probability of being seroprotected against A/H3N2 and B (sub)types; no other significant (SP against A/H1N1 and SCs against all three strains) results emerged (Supplementary Material, Table S7).

However, we did not retain the results obtained by Lee et al. [62], since RCTs (vitamin D supplementation vs. placebo or no treatment) and cohort studies based on the vitamin D serum concentration (deficient vs. normal) were pooled together.

Among the primary studies, four RCTs were identified; their sample sizes were, however, low. We did not find any significant (*p* > 0.27) association, regardless of the HAI measure used and the virus (sub)type (Table 3). Owing to the paucity of studies, no further analyses were conducted.

Observational studies on IV immunogenicity in vitamin D-deficient patients versus controls with normal concentrations did not usually reveal a significant difference. We decided against pooling since the available studies used different cut-offs to define vitamin D deficiency.

### 3.7. Immunosuppressive Conditions

The category of any immunosuppressive condition was the most populated: as mentioned above, a total of 17 SRs/SRMAs on this topic were included in the qualitative assessment.

A total of 96 pooled estimates (immunosuppressed patients vs. healthy controls) based on at least two studies were extracted from the nine SRMAs; the number of pooled studies ranged from 2 to 50, and 58% (*N* = 56) of the meta-analytical estimates reported a *p* < 0.05. Of the latter, all but one (*N* = 55) reported a negative effect of immunosuppression on the SC and/or SP against influenza (Supplementary Material, Table S8).

From the included SRs without MAs, the following qualitative evidence synthesis was drawn. Agarwal et al. [43] concluded that approximately 50% of the primary studies included had indicated a negative effect of IV in patients using immunosuppressive medications. Three SRs [46,51,60] involving cancer patients (either pediatric or adult) advocated the use of IVs in such patients, although uncertainty regarding the negative effect of the condition was substantial. Finally, the SR by Sousa et al. [59] on the effect of rheumatic diseases on IV immunogenicity concluded that the biological therapy of rheumatic diseases could hamper the immune response, but that IV could be beneficial in such patients.

Among the SRs/SRMAs that covered a similar topic, the observed corrected covered areas varied greatly. Indeed, the corrected covered area of three SRMAs on systemic lupus erythematosus [54,55,56] and another three SRMAs on rheumatoid arthritis [47,57,63] showed very high corrected covered areas (73.7% and 21.1%, respectively), while three SRs on cancer patients [46,51,60] and two SRMAs on transplant patients [45,52] showed substantially lower corrected covered areas, which were deemed “moderate” (6.6% and 8.9%, respectively).

Table 4 summarizes the evidence synthesis on the effect of any immunosuppressive condition on IV-induced immunogenicity. The total number of studies included varied from 69 to 116, and the corresponding number of cases ranged from 3720 to 8673. As expected, the highest number of studies involved A/H1N1, since several studies were conducted during the last H1N1pdm09 pandemic, when the monovalent vaccine was used. Despite the large patient numbers and effect sizes, the CES assigned was generally of class II (exception: SP and post-vaccination HAI titer for type B, which were assigned to class III). This was primarily attributed to the relatively high heterogeneity observed and the consequent large 95% prediction intervals, which downgraded the CES (Table 4).

With regard to post-vaccination HAI GMT titers, we conducted a sensitivity analysis after excluding studies with imputed SDs. Although k dropped significantly, the Hedges’ gs observed were in line with the main analysis (Table 4) and no substantial changes occurred; the CES grouping did not change (Supplementary Material, Table S9).

In order to explain the observed heterogeneity reported in Table 4, we conducted a series of meta-regression analyses (Supplementary Material, Tables S10–S12). The following variables were explored: year of publication (<2000 vs. ≥2000), total study sample (<100 vs. ≥100), population age (children vs. adults), a categorical variable of the virus (sub)type (A/H1N1, A/H3N2 and B, where A/H1N1 is the reference category) and a categorical variable of immunosuppressive condition. This last category was classified post-hoc in order to ensure a significant number of observations for each category. The classification was: transplant patients (reference category), cancer, HIV, rheumatic diseases and other/mixed conditions. In the fully adjusted models, only patients with rheumatic diseases generally showed a better response than transplant recipients, although the regression coefficients reached an *α* < 0.05 only with regard to the post-vaccination HAI titer (Supplementary Material, Tables S10–S12).

We then conducted several subgroup analyses according to the type of immunosuppression. As summarized in Table 5, most pooled estimates were statistically significant. However, although the ESs were usually large, the overall CES was often of class IV, as it was driven by the total sample size. Indeed, a class-I CES was assigned only to some pooled estimates that regarded the subtype A/H1N1. In any case, these subgroup analyses allowed us to reduce heterogeneity in several instances. A full description of the subgroup analyses conducted is reported in Supplementary Material, Tables S13–S19.

### 3.8. Diabetes Mellitus

Although diabetes mellitus may be regarded as an immunosuppressive condition, we decided to present it separately, owing to its enormous public health burden. An industry-sponsored SR by Dos Santos et al. [61] included a total of six IV-related immunogenicity studies, four of which included healthy subjects as controls. It was generally concluded that diabetes mellitus did not negatively interfere with IV-induced immunogenicity.

Owing to the different study populations and designs of the few studies available, no meta-analysis was re-performed.

### 3.9. Physical Exercise

The modifying effects of acute and chronic exercise were reviewed by Pascoe et al. [49]. Evidence of the effect of acute exercise on the IV-induced response was scant and restricted to younger adults. Single studies tested different hypotheses (e.g., the acute-stress immune-enhancement hypothesis, or an acute inflammatory response following local muscle damage resulting from vaccination) and yielded conflicting results. This latter effect could be ascribed to different sex-related mechanisms, such as exercise-sex interaction [49]. No meta-analysis on the effect of acute exercise on IV-induced immunogenicity was deemed to be of public health interest.

The studies on chronic exercise were more homogeneous in terms of both study populations (usually the elderly) and results; however, the study designs differed (RCTs and cross-sectional studies). As pre-specified in the “Materials and Methods” section, we excluded cross-sectional studies from any interpretation. Most studies found a positive correlation between chronic exercise and the immune response to IV [49].

In the present study, it was possible to pool SPs and post-vaccination HAI GMTs from three small RCTs involving older adults. SCs could not be pooled. As shown in Table 6, there was a significant association between chronic exercise and SPs against A/H1N1 and A/H3N2. Owing to the small sample sizes, the CES assigned was class IV. No other significant associations emerged.

### 3.10. Latent Cytomegalovirus Infection

A recent SRMA by van den Berg [67] revealed an unclear relationship between CMV seropositivity and IV-induced response in both adults and the elderly. In the MA, the OR of SC against any virus (sub)type did not reach an *α* < 0.05 (OR = 0.65 (95%: 0.40, 1.08); *p* = 0.11); heterogeneity was moderate (*I*^2^ = 33.2%). A subgroup analysis by age (adults <60 years and older adults ≥60 years) showed similar results [adults: OR = 0.41 (95% CI: 0.11, 1.45); older adults: OR = 0.57 (95% CI: 0.26, 1.25)].

In keeping with our aims, we re-performed the meta-analysis by single virus (sub)type. No significant association was found in either the main (Table 7) or subgroup analysis by age (Supplementary Material, Table S20), independently from the virus (sub)type and age-class.

### 3.11. Psychological Stress

An SRMA by Pedersen et al. [41] investigated the potential role of psychological stress on IV-induced immunogenicity. Stressful conditions were found to be associated with a statistically lower immune response. However, a few observations should be made on the pooled estimates by Pedersen et al. [41]. First, different serological assays (HAI and ELISA) were pooled together. Second, no distinction was made among the three vaccine components. Third, the ES used was the so-called effect size correlation coefficient, the interpretation of which is similar to Pearson’s r. For instance, a statistically significant negative effect size correlation coefficient indicates that a given stress condition is associated with lower antibody concentrations. The authors found that chronic stress (*p* = 0.001), life events (*p* = 0.0001) and perceived stress (*p* = 0.02) were all associated with a lower immune response; the effect size was, however low, with a correlation coefficient of −0.18 for all three groups of stress condition. Similar results were obtained in the analysis by age-group. The meta-analytical estimates extracted are reported in Supplementary Material, Table S21.

We decided not to re-perform a meta-analysis for several reasons. First, several studies reported the outcome of SC and/or SP for more than one (and sometimes all) of the vaccine strains together (and not separately by strain). Second, only a few studies reported raw data of interest; most used alternative measures and/or effect sizes (e.g., correlation and regression coefficients, *η*^2^ etc.). Third, it was difficult to categorize stressors. In any case, even if the meta-analysis had been re-performed, the overall CES would have been of class IV at the most, given the small sample sizes.

### 3.12. Evidence Mapping

**Figure 3:**
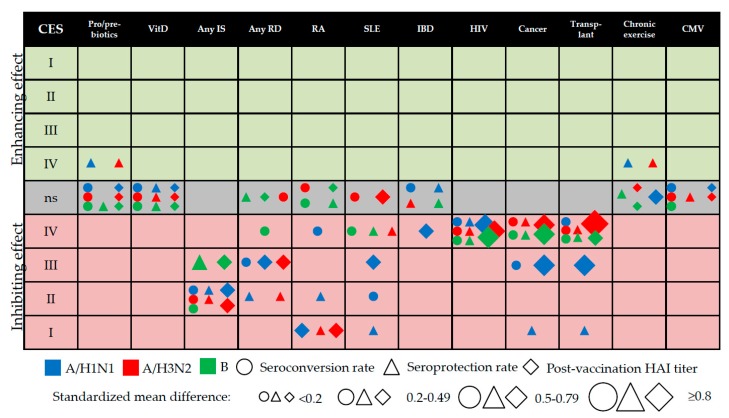
Bubble plot of the cumulative evidence synthesis (CES), by class, direction, (sub)type, serological parameter. The “ns” (non-significant) category stands for a non-significant effect (*p* > 0.05) of a given health condition; CMV: cytomegalovirus; HAI: hemagglutination-inhibition assay; HIV: human immunodeficiency virus; IBD: inflammatory bowel disease; IS: immunosuppression; RA: rheumatoid arthritis; RD; rheumatic disease; SLE: systemic lupus erythematosus: VitD: vitamin D supplementation.

A total of 97 meta-analytical estimates obtained were mapped in a single bubble plot (Figure 3). While constructing the bubble plot, we realized that the overall CES might not correspond exactly to the magnitude of the observed effect size. This non-correspondence was primarily driven by the total sample size. For instance, although the effect sizes regarding HIV were large, the overall CES was only of class IV, given *N* < 1000. In order to contextualize this, we first transformed the binary outcomes of SC/SP to the standardized mean difference (also known as Cohen’s d), by using the following formula [68]: d = ln(OR)∙∛/π, where π is a constant approximately equal to 3.14. This transformation was necessary in order to display binary (SCs and SPs) and continuous outcomes (difference in the post-vaccination HAI titers) on the same graph.

As shown in Figure 3, most host factors studied exerted a negative effect on IV-induced immunogenicity. Only four pooled estimates were associated with an enhancing effect: this was the case of SPs against A/H1N1 and A/H3N2 among the users of pre/pro/symbiotics and the physically active elderly. These estimates, however, were assigned a CES of only IV.

The distribution of single CESs and average modules of ESs is reported in Supplementary material, Table S22. As expected (since many studies were conducted during the last 2009 pandemic, when the monovalent A/H1N1pdm09 vaccine was used), most CES I pooled estimates were recorded only for A/H1N1. The omnibus analysis of variance (ANOVA) test did not show any difference between the observed |d| and virus (sub)type (F_2,94_ = 0.59, *p* = 0.56). By contrast, there was some difference between the ES observed and the CES assigned (F_4,92_ = 5.18, *p* = 0.0008).

## 4. Discussion

In this study, we examined the available systematic evidence on the relationship between several host-related factors (both modifiable and unmodifiable) and IV-induced immunogenicity; the analysis was made from both the qualitative and quantitative points of view. The increasing number of SRs/SRMAs on the same/similar topics and performed by different research groups using different systematic approaches may confound the decision-making of the principal stakeholders, including, for example, clinicians, reimbursement agencies and the pharmaceuticals industry. To overcome this problem, the emerging field of umbrella reviews [26] aims to synthetize the available systematic evidence. In addition to providing a standard representation of pooled estimates, the present research tried to classify this evidence in terms of CESs and to map the output of interest in a single figure (see Figure 3). The diversified categorization strategy used to map the evidence, together with the tabulated data presented in both the main text and the Supplementary Material, also allowed us to identify some evidence gaps.

Regarding external validity, our pooled estimates were generally in line with those previously published. The main exception concerned the use of pre-/pro-/symbiotics, in that our estimates were more conservative and usually non-significant. The most probable explanation for this is that different extraction modalities and statistical approaches were used. Moreover, as noted above, even the four significant estimates regarding the use of pre-/pro-/symbiotics established in this paper may be prone to the so-called “industry sponsorship” bias; these data should, therefore, be interpreted with caution.

The present study found that several (but not all) immunosuppressive conditions were associated with a significantly lower immune response to IV. Several parameters analyzed that regarded some immunosuppressive conditions, such as HIV, transplantation and cancer, were assigned only class-IV CESs, despite their moderate-to-high effect sizes; this was primarily the case of A/H3N2 and B viruses. The most obvious reason for this is that the total number of cases in which these parameters were investigated was often <1000; indeed, CES classes I–III require a total sample size of at least 1000 cases [38]. The parameters regarding the subtype A/H1N1 were less affected by this limitation since many primary studies investigated the immunogenicity of monovalent pandemic H1N1pdm09 vaccines. Indeed, further larger-scale studies on several immunosuppressive diseases are needed in order to upgrade the CESs observed. Moreover, the residual heterogeneity observed (after performing subgroup/meta-regression analyses) could be due to different treatment regimens in the same category of disease. Indeed, we included papers on the same immunosuppressive category published in the range 1970s–2010s—in these 40 years, the standard of care has changed radically [69,70]. In the future, more detailed analyses (e.g., by medicine type) will be needed. Our research group is now striving to identify the most appropriate way to study this issue.

The levels of available evidence regarding intravenous drug use, genetic polymorphisms, diabetes mellitus, acute physical activity, psychological stress and previous BCG vaccination were not included in the overall evidence synthesis reported in Figure 3, owing to the paucity of primary studies available and/or overlap between the categories included. These factors need to be (re)-examined in the future, given their enormous public health burden.

Our results may have several public health implications. According to the latest report on seasonal IVs, issued by the European Centre for Disease Prevention and Control [71], all Member States recommend vaccination for subjects suffering from immunosuppression due to disease or treatment and hematologic/metabolic disorders. On the one hand, these populations are at increased risk of developing severe and life-threatening disease [1]; on the other, the immune response to IV may be highly compromised (Figure 3). There is some evidence [72,73,74,75] that alternative (so-called “enhanced”) IVs, including adjuvanted and high-dose (quadruple antigen content) formulations, produce a more robust immune response than conventional IVs in immunosuppressed individuals. However, these vaccines are currently indicated for people aged 65 years or more [4]. Alternatively, a two-dose regimen could be considered. However, the effect of a booster dose on IV-induced immunogenicity is controversial. For instance, an SRMA by Liao et al. [76] found that a booster did not have any beneficial effect on the immune response in patients on hemodialysis or peritoneal dialysis or in renal transplant recipients. Moreover, the novel egg-independent technologies with several potential advantages are becoming increasingly common [77,78] and should be further investigated also from the immunogenicity point of view.

While the IV formulation and dose regimen in immunocompromised individuals are still to be clarified, the public health strategy aimed at protecting this vulnerable population indirectly is to be pursued. Indeed, vaccinating household contacts and caregivers (including healthcare workers) is highly recommended [79,80].

Regarding the so-called “natural adjuvants”, including the concomitant use of pre-/pro-/symbiotics, vitamin D supplements or moderate physical exercise, we found almost no effect on the IV-induced immune response. The few statistically significant pooled estimates had low ESs (all CESs IV) and were therefore of doubtful practical/public health significance.

Together with its strengths, several limitations of the present study must be acknowledged. Given the umbrella review approach, three major categories of systematic error could occur, namely (i) bias regarding our own methodology; (ii) bias regarding the SR/SRMAs included and (iii) bias regarding primary studies.

Considering the first point, we tried to create a more sensitive (and therefore less specific) search script, in order to identify as many as possible of the publicly available SRs. The script created for the automatic search strategy was, in our opinion, sufficient to discover most publicly available SRs/SRMAs—indeed, the manual search did not produce any further results. However, we acknowledge that some SRs that were not dubbed as such by their authors might have been missed by our search strategy.

The second limitation, in our view, is the most challenging, since the reporting quality of the SRs/SRMAs included was generally moderate-to-low, as per the AMSTAR-2 checklist. One explanation may lie in the fact that several SRs/SRMAs were published before the publication of AMSTAR-2 [30]. Indeed, if we estimate a two-year lag (as the time needed for the authors to acquaint themselves with the new “quality” scale for their SRs/SRMAs protocols and for peer-review and proof correction) between the publication year of the SRs/SRMAs included and the paper by Shea et al. published in 2017 [30], we can see that only 1/28 of the SRs/SRMAs included was published in 2019. Notably, this paper [67] had a relatively high AMSTAR-2 rating (75% of “Yes” ratings). However, as mentioned in the Methods section, the original AMSTAR scale, which was published in 2007 [81], included most of the items contained in the newer version. Therefore, the novelty of AMSTAR-2 is unlikely to have been the main driver of the suboptimal reporting quality of the SRs/SRMAs included.

Again with regard to the second limitation, we must acknowledge that most primary research studies were included on the basis of existing SRs/SRMAs (which is essential in an umbrella review). We strove to mitigate this limitation by performing an updated search by means of the same search strategy and study inclusion criteria as the original SR/SRMAs. We did not, however, test the sensitivity/specificity parameters of the single search strategies, since this was beyond the scope of our study.

Third, we did not investigate any risk of bias among single primary research studies included in the present analysis. Again, this went well beyond our aims. Indeed, as demonstrated by the AMSTAR-2 checklist, only 16 of 28 (57%) SR/SRMAs used a satisfactory technique to assess the RoB.

We have some suggestions for future research. First of all, authors of papers on IV-induced immunogenicity should be aware of the currently used terminology, i.e., immunogenicity, efficacy and effectiveness. For instance, we found several studies (even those conducted in the past few years) dealing with CoPs that were entitled “efficacy” studies, without the surrogate nature of the outcome of protection being mentioned in the abstract/title. This fact may have altered the search output of the SRs/SRMAs included, as well as slowing down the selection/abstracting process. We, therefore, invite researchers outside IV-related topics to adopt a single terminology, such as, for example, that proposed by the US Centers of Disease Control and Prevention [82].

In conclusion, the present research mapped the available evidence on several modifiers of the IV-induced vaccine response. While the inhibiting effect of several immunosuppressive host factors was evident, the enhancing effect of pro/pre/symbiotics and chronic physical exercise was doubtful and virus type-specific (A but not B); the overall CES was only IV. In other words, the included SR/SRMAs on host factors with potentially enhancing effect on the IV-induced immunogenicity suffered from several limitations (e.g., a low number of included primary studies with a few participants with well-known effects on statistical power in the pooled estimates; possibility of the “industry sponsorship” bias since the funding source of the primary trials was not investigated; etc.). Therefore, future well-designed RCTs or observational studies on the effect of this “natural adjuvants” are needed. On the other hand, we discovered that the pooled effect sizes observed may not exactly correspond to the CES assigned. This means that further studies are needed in order to upgrade the overall CES grading system.

Furthermore, nowadays the field of vaccinology is still empirical in several aspects [83]. The current limited knowledge into qualitative and quantitative paradigms of the immune response generated by vaccines is a serious barrier to understanding poor vaccine immunogenicity in a plethora of physiological and pathological conditions. Today, emerging scientific lines propose a personalized approach to the practice of vaccinology, as it happens for other healthcare fields [83,84]. Studying the host-related correlates of influenza vaccine-induced immune response could contribute to the production of new personalized vaccines and to the development of new patient-oriented vaccination strategies in a value-based public health perspective.

## Figures and Tables

**Table 1:** Main characteristics of the systematic reviews and/or meta-analyses included.

Study [Ref]	Year	Factor(s) Assessed	Type of Study	Population ^†^	*k* ^†^	Meta-Analysis Performed	AMSTAR-2 ^‡^
Baral [40]	2007	Intravenous drug use	Obs	Adults	2	No	2/2/8/4
Pedersen [41]	2009	Psychological stress	Obs	All	13	Yes	7/2/7/0
Beck [42]	2011	Immunosuppression of any etiology	RCT, obs	All	209	Yes	12/0/4/0
Agarwal [43]	2012	Immunosuppressive drugs	RCT, obs	All	11	No	3/1/8/4
Beck [44]	2012	Immunosuppression by etiology	RCT, obs	All	209	Yes	12/0/4/0
Eckerle [45]	2013	Solid organ transplants	RCT, obs	All	36	Yes	6/1/9/0
Goossen [46]	2013	Chemotherapy in cancer patients	RCT, CCT	Children	9	No	10/0/2/4
Hua [47]	2014	Antirheumatic drugs in rheumatoid arthritis patients	Obs	Adults	7	Yes	5/2/9/0
McMahan [48]	2014	Biological and non-biological drugs in rheumatic disease patients	Unclear	All	18	No	1/0/11/4
Pascoe [49]	2014	Acute and chronic physical exercise	RCT, obs	All	15	No	5/1/6/4
Posteraro [50]	2014	Genetic variations	Obs	Adults	1	Yes	7/1/8/0
Shehata [51]	2014	Cancer patients on systemic treatment	Unclear	All	16	No	2/0/10/4
Karbasi-Afshar [52]	2015	Transplant recipients	Obs	Unclear	15	Yes	1/0/15/0
Nguyen [53]	2015	Immunosuppressive drugs in inflammatory bowel disease patients	Obs	All	2	Yes	2/3/11/0
Huang [54]	2016	Systemic lupus erythematosus	Obs	All	15	Yes	11/1/4/0
Liao [55]	2016	Systemic lupus erythematosus	Obs	All	18	Yes	8/2/6/0
Pugès [56]	2016	Systemic lupus erythematosus	Obs	Adults	17	Yes	10/1/5/0
Huang [57]	2017	Rheumatoid arthritis	Obs	Adults	13	Yes	8/1/7/0
Lei [58]	2017	Probiotics, prebiotics and symbiotics	RCT	Adults	20	Yes	9/1/6/0
Sousa [59]	2017	Rheumatic diseases	RCT, obs	Children	11	No	3/0/9/4
Vollaard [60]	2017	Solid tumor patients on chemotherapy	Obs	Adults	20	No	1/0/15/0
Dos Santos [61]	2018	Diabetes mellitus	RCT, obs	All	15	No	6/0/6/4
Lee [62]	2018	Vitamin D deficiency/supplementation	RCT	All	9	Yes	6/2/8/0
Subesinghe [63]	2018	Antirheumatic drugs in rheumatoid arthritis patients	Obs	Adults	7	Yes	9/1/6/0
Yeh [64]	2018	Probiotics, prebiotics and symbiotics	RCT	Adults	20	Yes	8/2/6/0
Zimmermann [65]	2018	Probiotics	RCT	Adults	12	No	3/1/8/4
Zimmermann [66]	2018	BCG vaccination	RCT, CCT	Adults	3	No	3/1/8/4
van den Berg [67]	2019	Latent CMV infection	RCT, obs	All	15	Yes	12/0/4/0

^†^ Considering only studies on influenza vaccines (if a systematic review also considered other vaccines); ^‡^ Results are reported as Yes/Partial yes/No/Not applicable; AMSTAR: measurement tool for assessing systematic reviews; BCG: Bacillus Calmette–Guérin; CCT: controlled clinical trial; CMV: cytomegalovirus; IBD: inflammatory bowel disease; RCT: randomized controlled trial; Obs: observational study.

**Table 2:** Summary evidence of the effect of using probiotics, prebiotics or symbiotics to enhance the influenza vaccine-induced immune response, by immunogenicity parameter and viral (sub)type.

Parameter	A/H1N1	A/H3N2	B
Seroconversion rate			
*k*	10	8	9
*N*	277/274	235/229	266/250
OR RE (95% CI)	1.55 (0.86, 2.90)	1.34 (0.72, 2.50)	1.14 (0.75, 1.74)
*p* RE	0.14	0.35	0.54
95% PI	0.38, 6.50	0.36, 5.00	0.75, 1.74
OR FE (95% CI)	1.42 (0.96, 2.09)	1.12 (0.75, 1.66)	1.14 (0.75, 1.74)
*p* FE	0.075	0.58	0.54
*I*^2^, %	49.6	49.0	0
*τ* ^2^	0.42	0.35	0
SSE, *p*	0.49	NA	NA
LS	No	No	No
CES	ns	ns	ns
Seroprotection rate			
*k*	13	11	11
*N*	845/855	805/812	800/810
OR RE (95% CI)	1.68 (1.02, 2.75)	1.93 (1.08, 3.44)	0.94 (0.73, 1.23)
*p* RE	0.040	0.026	0.66
95% PI	0.47, 5.98	0.64, 3.13	0.73, 1.23
OR FE (95% CI)	1.25 (0.98, 1.59)	1.94 (1.20, 3.13)	0.94 (0.73, 1.23)
*p* FE	0.067	0.006	0.66
*I*^2^, %	56.2	24.9	0
*τ* ^2^	0.36	0.23	0
SSE, *p*	0.18	0.58	0.38
LS	No	No	No
CES	IV	IV	ns
Post-vaccination HAI titer			
*k*	11	10	10
*N*	399/398	380/374	380/374
*g* RE (95% CI)	0.05 (−0.09, 0.19)	0.05 (−0.10, 0.19)	0.00 (−0.15, 0.14)
*p* RE	0.49	0.53	0.96
95% PI	−0.09, 0.19	−0.10, 0.19	−0.15, 0.14
*g* FE (95% CI)	0.05 (−0.09, 0.19)	0.05 (−0.10, 0.19)	0.00 (−0.15, 0.14)
*p* FE	0.49	0.53	0.96
*I*^2^, %	0	0	0
*τ* ^2^	0	0	0
SSE, *p*	0.84	0.78	0.009
LS	No	No	No
CES	ns	ns	ns

CES: cumulative evidence synthesis class; CI: confidence interval; FE: fixed-effects model; HAI: hemagglutination-inhibition; LS: the largest study has a statistically significant effect size; OR: odds ratio; PI: prediction interval; RE: random-effects model; SSE: small-study effect test.

**Table 3:** Summary evidence of the effect of vitamin D supplementation in order to enhance the influenza vaccine-induced immune response, by immunogenicity parameter and viral (sub)type.

Parameter	A/H1N1	A/H3N2	B
Seroconversion rate			
*k*	3	3	3
*N*	176/276	176/276	176/276
OR RE (95% CI)	0.79 (0.52, 1.20)	1.02 (0.69, 1.51)	0.93 (0.57, 1.52)
*p* RE	0.27	0.94	0.77
95% PI	0.52, 1.20	0.69, 1.51	0.57, 1.52
OR FE (95% CI)	0.79 (0.52, 1.20)	1.02 (0.69, 1.51)	0.93 (0.57, 1.52)
*p* FE	0.27	0.94	0.77
*I*^2^, %	0	0	0
*τ* ^2^	0	0	0
SSE, *p*	NA	NA	NA
LS	No	No	No
**CES**	ns	ns	ns
Seroprotection rate			
*k*	3	3	3
*N*	176/276	176/276	176/276
OR RE (95% CI)	0.85 (0.51, 1.41)	0.98 (0.60, 1.58)	0.75 (0.44, 1.28)
*p* RE	0.53	0.92	0.29
95% PI	0.51, 1.41	0.60, 1.58	0.44, 1.28
OR FE (95% CI)	0.85 (0.51, 1.41)	0.98 (0.60, 1.58)	0.75 (0.44, 1.28)
*p* FE	0.53	0.92	0.29
*I*^2^, %	0	0	0
*τ* ^2^	0	0	0
SSE, *p*	NA	NA	NA
LS	No	No	No
CES	ns	ns	ns
Post-vaccination HAI titer			
*k*	3	3	3
*N*	154/153	154/153	154/153
*g* RE (95% CI)	0.07 (−0.17, 0.30)	−0.05 (−0.27, 0.17)	0.02 (−0.21, 0.24)
*p* RE	0.58	0.66	0.89
95% PI	−0.18, 0.31	−0.27, 0.17	−0.21, 0.24
*g* FE (95% CI)	0.06 (−0.16, 0.29)	−0.05 (−0.27, 0.17)	0.02 (−0.21, 0.24)
*p* FE	0.57	0.66	0.89
*I*^2^, %	4.2	0	0
*τ* ^2^	0	0	0
SSE, *p*	NA	NA	NA
LS	No	No	No
CES	ns	ns	ns

CES: cumulative evidence synthesis class; CI: confidence interval; FE: fixed-effects model; HAI: hemagglutination-inhibition; LS: the largest study has a statistically significant effect size; OR: odds ratio; PI: prediction interval; RE: random-effects model; SSE: small-study effect test.

**Table 4:** Summary evidence of the effect of immunosuppressive conditions on the influenza vaccine-induced immune response, by immunogenicity parameter and viral (sub)type.

Parameter	A/H1N1	A/H3N2	B
Seroconversion rate			
*k*	116	94	85
*N*	8673/4638	4193/3023	3888/2944
OR RE (95% CI)	0.50 (0.42, 0.59)	0.51 (0.41, 0.63)	0.53 (0.44, 0.64)
*p* RE	2∙10^−15^	2∙10^−10^	4∙10^−11^
95% PI	0.13, 1.99	0.11, 2.37	0.16, 1.75
OR FE (95% CI)	0.53 (0.49, 0.59)	0.56 (0.50, 0.62)	0.54 (0.48, 0.61)
*p* FE	<1∙10^−14^	<1∙10^−14^	<1∙10^−14^
*I*^2^, %	65.7	65.8	53.9
*τ* ^2^	0.49	0.60	0.36
SSE, *p*	0.30	0.30	0.75
LS	Yes	Yes	Yes
CES	II	II	II
Seroprotection rate			
*k*	102	76	75
*N*	8452/4272	3780/2605	3759/2505
OR RE (95% CI)	0.42 (0.35, 0.51)	0.35 (0.27, 0.45)	0.53 (0.41, 0.69)
*p* RE	<1∙10^−15^	1∙10^−15^	3∙10^−6^
95% PI	0.12, 1.54	0.08, 1.46	0.10, 2.76
OR FE (95% CI)	0.44 (0.39, 0.49)	0.38 (0.32, 0.45)	0.51 (0.44, 0.60)
*p* FE	<1∙10^−15^	<1∙10^−15^	<1∙10^−15^
*I*^2^, %	54.5	45.7	59.9
*τ* ^2^	0.43	0.52	0.69
SSE, *p*	>0.99	0.78	0.12
LS	Yes	Yes	Yes
CES	II	II	III
Post-vaccination HAI titer			
*k*	99	77	69
*N*	7909/4438	3889/2922	3720/2751
*g* RE (95% CI)	−0.36 (−0.45, −0.28)	−0.44 (−0.55, −0.34)	−0.34 (−0.43, −0.24)
*p* RE	<1∙10^−15^	2∙10^−15^	2∙10^−12^
95% PI	−1.05, 0.32	−1.25, 0.36	−0.94, 0.26
*g* FE (95% CI)	−0.33 (−0.37, −0.29)	−0.43 (−0.49, −0.38)	−0.32 (−0.38, −0.27)
*p* FE	<1∙10^−15^	<1∙10^−15^	<1∙10^−15^
*I*^2^, %	73.8	75.0	63.6
*τ* ^2^	0.12	0.17	0.09
SSE, *p*	0.17	0.64	0.42
LS	Yes	Yes	No
CES	II	II	III

CES: cumulative evidence synthesis class; CI: confidence interval; FE: fixed-effects model; HAI: hemagglutination-inhibition; LS: the largest study has a statistically significant effect size; OR: odds ratio; PI: prediction interval; RE: random-effects model; SSE: small-study effect test.

**Table 5:** Summary evidence of the effect of single immunosuppressive conditions on the influenza vaccine-induced immune response, by immunogenicity parameter and viral (sub)type.

Virus	Parameter		Any RD	RA	SLE	IBD	HIV	Cancer	Transplantation
A/H1N1	SC	OR	0.57	0.64	0.35	0.54	0.51	0.49	0.49
		CES	III	IV	II	ns	IV	III	IV
	SP	OR	0.47	0.39	0.38	0.80	0.35	0.28	0.28
		CES	II	II	I	ns	IV	I	I
	HAI titer	*g*	−0.21	−0.37	−0.42	−0.30	−0.51	−0.61	−0.61
		CES	III	I	III	IV	IV	III	III
A/H3N2	SC	OR	0.78	0.97	0.55	NA	0.46	0.44	0.35
		CES	ns	ns	ns	NA	IV	IV	IV
	SP	OR	0.40	0.37	0.39	0.74	0.21	0.37	0.26
		CES	II	IV	IV	ns	IV	IV	IV
	HAI titer	*g*	−0.26	−0.26	−0.23	NA	−0.77	−0.54	−0.89
		CES	III	IV	ns	NA	IV	IV	IV
B	SC	OR	0.75	0.72	0.57	NA	0.37	0.41	0.54
		CES	IV	ns	IV	NA	IV	IV	IV
	SP	OR	0.76	0.84	0.60	1.12	0.33	0.46	0.40
		CES	ns	ns	IV	ns	IV	IV	IV
	HAI titer	*g*	−0.05	−0.11	NA	NA	−0.54	−0.54	−0.49
		CES	ns	ns	NA	NA	IV	IV	IV

CES: cumulative evidence synthesis class; HAI: hemagglutination-inhibition; HIV: human immunodeficiency virus; IBD: inflammatory bowel disease; ns: CES “non-significant”, i.e., the pooled *p* > 0.05; OR: odds ratio; RA: rheumatoid arthritis; RD: rheumatic disease; SC: seroconversion rate; SLE: systemic lupus erythematosus; SP: seroprotection rate.

**Table 6:** Summary evidence of the effect of chronic physical exercise on influenza vaccine-induced immune response in the elderly, by immunogenicity parameter and viral (sub)type.

Parameter	A/H1N1	A/H3N2	B
Seroprotection rate			
*k*	3	3	3
*N*	115/106	115/106	115/106
OR RE (95% CI)	2.70 (1.45, 5.02)	1.95 (1.11, 3.43)	1.31 (0.74, 2.30)
*p* RE	0.0017	0.020	0.36
95% PI	1.45, 5.02	1.11, 3.43	0.74, 2.30
OR FE (95% CI)	2.70 (1.45, 5.02)	1.95 (1.11, 3.43)	1.31 (0.74, 2.30)
*p* FE	0.0017	0.020	0.36
*I*^2^, %	0	0	0
*τ* ^2^	0	0	0
SSE, *p*	NA	NA	NA
LS	Yes	Yes	No
CES	IV	IV	ns
Post-vaccination HAI titer			
*k*	2	2	2
*N*	88/83	88/83	88/83
*g* RE (95% CI)	0.28 (−0.39, 0.96)	0.19 (−0.12, 0.49)	−0.07 (−0.37, 0.23)
*p* RE	0.41	0.23	0.65
95% PI	−0.75, 1.32	−0.12, 0.49	−0.37, 0.23
*g* FE (95% CI)	0.13 (−0.18, 0.43)	0.19 (−0.12, 0.49)	−0.07 (−0.37, 0.23)
*p* FE	0.42	0.23	0.65
*I*^2^, %	63.4	0	0
*τ* ^2^	0.16	0	0
SSE, *p*	NA	NA	NA
LS	No	No	No
CES	ns	ns	ns

CES: cumulative evidence synthesis class; CI: confidence interval; FE: fixed-effects model; HAI: hemagglutination-inhibition; LS: the largest study has a statistically significant effect size; OR: odds ratio; PI: prediction interval; RE: random-effects model; SSE: small-study effect test.

**Table 7:** Summary evidence of the effect of latent cytomegalovirus (CMV) infection on the influenza vaccine-induced immune response, by immunogenicity parameter and viral (sub)type.

Parameter	A/H1N1	A/H3N2	B
Seroconversion rate			
*k*	6	7	9
*N*	192/83	670/203	99/43
OR RE (95% CI)	0.46 (0.14, 1.56)	1.05 (0.74, 1.49)	0.64 (0.27, 1.56)
*p* RE	0.21	0.79	0.33
95% PI	0.03, 6.65	0.74, 1.49	0.27, 1.56
OR FE (95% CI)	0.58 (0.29, 1.16)	1.05 (0.74, 1.49)	0.64 (0.27, 1.56)
*p* FE	0.12	0.79	0.33
*I*^2^, %	65.7	0	0
*τ* ^2^	1.47	0	0
SSE, *p*	NA	NA	NA
LS	Yes	No	No
CES	ns	ns	ns
Seroprotection rate			
*k*	NA	5	NA
*N*	NA	616/191	NA
OR RE (95% CI)	NA	1.08 (0.76, 1.54)	NA
*p* RE	NA	0.42	NA
95% PI	NA	0.76, 1.54	NA
OR FE (95% CI)	NA	1.08 (0.76, 1.54)	NA
*p* FE	NA	0.42	NA
*I*^2^, %	NA	0	NA
*τ* ^2^	NA	0	NA
SSE, *p*	NA	NA	NA
LS	NA	No	NA
CES	NA	ns	NA
Post-vaccination HAI titer			
*k*	7	7	NA
*N*	371/221	716/260	NA
*g* RE (95% CI)	−0.25 (−0.58, 0.08)	−0.06 (−0.22, 0.11)	NA
*p* RE	0.14	0.50	NA
95% PI	−0.99, 0.50	−0.31, 0.20	NA
*g* FE (95% CI)	−0.13 (−0.31, 0.04)	−0.06 (−0.20, 0.09)	NA
*p* FE	0.13	0.45	NA
*I*^2^, %	65.0	20.3	NA
*τ* ^2^	0.12	0.01	NA
SSE, *p*	NA	NA	NA
LS	No	No	NA
CES	ns	ns	NA

CES: cumulative evidence synthesis class; CI: confidence interval; FE: fixed-effects model; LS: the largest study has a statistically significant effect size; OR: odds ratio; PI: prediction interval; RE: random-effects model; SSE: small-study effect test.

## Supplementary Materials

The following are available online at https://www.mdpi.com/2076-393X/7/4/215/s1, Table S1: List of excluded studies, with reasons; Table S2: AMSTAR-2 (measurement tool for assessing systematic reviews, version 2) ratings, by paper and item; Table S3: Pooled estimates extracted from the available meta-analyses of the effect of probiotic, prebiotic or symbiotic use in order to enhance the influenza vaccine-induced immune response; Table S4: Subgroup analysis (by supplement type and age-class) of the summary evidence of the effect of probiotic, prebiotic or symbiotic use on seroconversion rates; Table S5: Subgroup analysis (by supplement type and age-class) of the summary evidence of the effect of probiotic, prebiotic or symbiotic use on seroprotection rates; Table S6: Subgroup analysis (by supplement type and age-class) of the summary evidence of the effect of probiotic, prebiotic or symbiotic use on post-vaccination hemagglutination-inhibition titers; Table S7: Pooled estimates extracted from the available meta-analyses of the effect of vitamin D deficiency on the influenza vaccine-induced immune response; Table S8: Pooled estimates extracted from the available meta-analyses of the effect of immunosuppressive conditions on the influenza vaccine-induced immune response; Table S9: Sensitivity analysis (on excluding studies with imputed standard deviations) of the effect of any immunosuppressive condition on post-vaccination hemagglutination-inhibition titers; Table S10: Multivariable meta-regression analysis in order to predict the observed pooled estimates of seroconversion rates; Table S11: Multivariable meta-regression analysis in order to predict the observed pooled estimates of seroprotection rates; Table S12: Multivariable meta-regression analysis in order to predict the observed pooled estimates of post-vaccination hemagglutination-inhibition titers; Table S13: Summary evidence of the effect of rheumatic diseases on the influenza vaccine-induced immune response, by immunogenicity parameter and viral (sub)type; Table S14: Summary evidence of the effect of rheumatoid arthritis on the influenza vaccine-induced immune response, by immunogenicity parameter and viral (sub)type; Table S15: Summary evidence of the effect of systemic lupus erythematosus on the influenza vaccine-induced immune response, by immunogenicity parameter and viral (sub)type; Table S16: Summary evidence of the effect of inflammatory bowel disease on the influenza vaccine-induced immune response, by immunogenicity parameter and viral (sub)type; Table S17: Summary evidence of the effect of human immunodeficiency virus (HIV) infection on the influenza vaccine-induced immune response, by immunogenicity parameter and viral (sub)type; Table S18: Summary evidence of the effect of transplantation on the influenza vaccine-induced immune response, by immunogenicity parameter and viral (sub)type; Table S19: Summary evidence of the effect of cancer on the influenza vaccine-induced immune response, by immunogenicity parameter and viral (sub)type; Table S20: Subgroup analysis (by age-class) of the summary evidence of the effect of latent cytomegalovirus (CMV) infection on the influenza vaccine-induced immune response; Table S21: Pooled estimates extracted from the available meta-analyses of the effect of psychological stress on the influenza vaccine-induced immune response; Table S22: Distribution of cumulative evidence synthesis classes and mean Cohen’s *d*s, by viral (sub)type.
