# Supplementary material for: Mapping Host-Related Correlates of Influenza Vaccine-Induced Immune Response: An Umbrella Review of the Available Systematic Reviews and Meta-Analyses
Source: Vaccines (Basel). 2019 Dec 13;7(4):215. doi: 10.3390/vaccines7040215 (PMC6963823; doi:10.3390/vaccines7040215)
Supplement: Supplementary file 1 [file vaccines-07-00215-s001.pdf]

Table S1. List of excluded studies, with reasons

Study	Reason for exclusion
Seidman, 2012	No predefined control group
Beck, 2013	Commentary on two published systematic reviews (both were included)
Hua, 2013	Conference abstract without sufficient details
Ramos, 2015	Non-systematic nature of the review
La Torre, 2016	No predefined control group
Liao, 2016	No predefined control group
Bekkat-Berkani, 2017	No predefined control group
Chong, 2018	Comparison of different influenza vaccine types
Zhang, 2018	Comparison of different influenza vaccine types; no predefined control group

References:

Seidman, J.C.; Richard, S.A.; Viboud, C.; Miller, M.A. Quantitative review of antibody response to inactivated seasonal influenza vaccines. *Influenza Other Respir. Viruses* **2012**, *6*, 52–62.

Beck, C.R.; McKenzie, B.C.; Hashim, A.B.; Harris, R.C.; Zanuzdana, A.; Agboado, G.; Orton, E.; Béchard-Evans, L.; Morgan, G.; Stevenson, C.; et al. Influenza vaccination for immunocompromised patients: summary of a systematic review and meta-analysis. *Influenza Other Respir. Viruses*. **2013**, *7* Suppl 2, 72–75.

Hua, C.; Barnetche, T.; Combe, B.; Morel, J. Influence of methotrexate, anti-tnf and rituximab on the immune response to influenza and pneumococcal vaccines in patients with rheumatoid arthritis: a systematic literature review and meta-analysis. *Ann. Rheum. Dis*. **2013**, *72*, A408.

Ramos, I.; Fernandez-Sesma, A. Modulating the innate immune response to influenza A virus: Potential therapeutic use of anti-inflammatory drugs. *Front. Immunol*. **2015**, *6*, 361.

La Torre, G.; Mannocci, A.; Colamesta, V.; D'Egidio, V.; Sestili, C.; Spadea, A. Influenza and pneumococcal vaccination in hematological malignancies: a systematic review of efficacy, effectiveness, and safety. *Mediterr. J. Hematol. Infect. Dis*. **2016**, *1*, 8, e2016044.

Liao, Z.; Xu, X.; Liang, Y.; Xiong, Y.; Chen, R.; Ni, J. Effect of a booster dose of influenza vaccine in patients with hemodialysis, peritoneal dialysis and renal transplant recipients: A systematic literature review and meta-analysis. *Hum. Vaccin. Immunother.* **2016**, *12*, 2909–2915.

Bekkat-Berkani, R.; Wilkinson, T.; Buchy, P.; Dos Santos, G.; Stefanidis, D.; Devaster, J.M.; Meyer, N. Seasonal influenza vaccination in patients with COPD: a systematic literature review. *BMC Pulm. Med*. **2017**, *17*, 79.

Chong, P.P.; Handler, L.; Weber, D.J. A systematic review of safety and immunogenicity of influenza vaccination strategies in solid organ transplant recipients. *Clin. Infect. Dis*. **2018**, *66*, 1802–1811.

Zhang, W.; Sun, H.; Atiquzzaman, M.; Sou, J.; Anis, A.H. Cooper C. Influenza vaccination for HIV-positive people: Systematic review and network meta-analysis. *Vaccine* **2018**, *36*, 4077–4086.

Table S2. AMSTAR-2 (measurement tool for assessing systematic reviews, version 2) ratings, by paper and item.

Study [Ref]	Item															
	**1**	**2**	**3**	**4**	**5**	**6**	**7**	**8**	**9**	**10**	**11**	**12**	**13**	**14**	**15**	**16**
**Baral [40]**	–	–	–	+/-	–	+	–	+/-	–	–	0	0	–	0	0	+
**Pedersen [41]**	+	–	–	+/-	–	+	+	+	+/-	–	+	–	–	+	+	–
**Beck [42]**	+	–	+	+	+	+	–	+	+	–	+	–	–	+	+	+
**Agarwal [43]**	+	–	–	+/-	+	–	–	–	–	–	0	0	–	0	0	+
**Beck [44]**	+	–	+	+	+	+	–	+	+	–	+	–	–	+	+	+
**Eckerle [45]**	+	–	–	+/-	+	–	–	+	–	–	+	–	–	+	–	+
**Goossen [46]**	+	+	–	+	+	+	+	+	+	–	0	0	+	0	0	+
**Hua [47]**	+	+/-	–	+/-	–	+	–	–	+	–	+	–	–	+	–	–
**McMahan [48]**	–	–	–	–	–	–	–	–	+	–	0	0	–	0	0	+
**Pascoe [49]**	+	–	–	+/-	+	+	–	+	–	–	0	0	–	0	0	+
**Posteraro [50]**	+	–	–	+/-	+	+	–	+	+	–	+	–	–	–	–	+
**Shehata [51]**	–	–	–	–	–	+	–	–	–	–	0	0	–	0	0	+
**Karbasi-Afshar [52]**	–	–	–	–	–	–	–	–	+	–	–	–	–	–	–	+
**Nguyen [53]**	–	–	–	+/-	–	–	–	+/-	+/-	–	–	–	–	+	–	+
**Huang [54]**	+	–	–	+/-	+	+	–	+	+	–	+	+	+	+	+	+
**Liao [55]**	+	–	–	+/-	–	+	–	+/-	+	–	+	+	+	–	+	+
**Pugès [56]**	+	–	–	+/-	+	+	–	+	+	–	+	+	+	+	–	+
**Huang [57]**	+	–	–	+/-	+	+	–	+	+	–	+	–	–	–	+	+
**Lei [58]**	+	–	–	+/-	+	+	–	+	+	–	+	–	–	+	+	+
**Sousa [59]**	+	–	–	–	+	–	–	+	+	–	0	–	–	0	0	–
**Vollaard [60]**	–	–	–	–	–	–	–	–	+	–	0	0	–	0	0	+
**Dos Santos [61]**	+	–	–	+	+	–	–	+	+	–	0	0	+	0	0	–
**Lee [62]**	–	+/-	–	+/-	+	+	–	+	+	–	+	–	–	–	–	–
**Subesinghe [63]**	+	–	–	+/-	+	+	–	+	+	–	+	+	+	–	–	–
**Yeh [64]**	+	+/-	–	+/-	+	+	–	+	+	–	–	–	–	+	+	+
**Zimmermann [65]**	+	–	–	+/-	–	–	–	+	–	–	0	0	–	0	0	+
**Zimmermann [66]**	+	–	–	+/-	–	–	–	+	–	–	0	0	–	0	0	+
**van den Berg [67]**	+	–	–	+	+	+	–	+	+	–	+	+	+	+	+	+

The ratings are reported as: Yes (+); Partial yes (+/-); No (–) and Not applicable (0)

Table S3. Pooled estimates extracted from the available meta-analyses of the effect of probiotic, prebiotic or symbiotic use in order to enhance the influenza vaccine-induced immune response (all models are random-effects).

Study	Supplement type	Population	Virus	Parameter (ES)	*k*	ES	95% CI	*P*	I^2, %
Lei [58]	Pro/ prebiotics	Any	A/H1N1	SC (OR)	6	1.52	0.75, 3.09	0.25	51
Lei [58]				SP (OR)	7	1.83	1.19, 2.82	0.006	0
Yeh [64]				Δ mean titers (MD)	12	7.14	2.73, 11.55	0.002	96
Lei [58]		Adults		SC (OR)	2	0.62	0.18, 2.14	0.45	0
Lei [58]		Elderly		SC (OR)	3	2.93	1.47, 5.87	0.002	3
Lei [58]		Any	A/H3N2	SC (OR)	6	2.54	0.93, 6.91	0.07	83
Lei [58]				SP (OR)	7	2.85	1.59, 5.10	0.0004	0
Yeh [64]				Δ mean titers (MD)	12	17.19	3.39, 30.99	0.01	100
Lei [58]		Adults		SC (OR)	2	3.46	1.22, 9.83	0.02	0
Lei [58]		Elderly		SC (OR)	3	3.68	1.11, 12.25	0.03	75
Lei [58]		Any	B	SC (OR)	6	2.11	1.38, 3.21	0.0006	0
Lei [58]				SP (OR)	7	0.99	0.65, 1.52	0.97	0
Yeh [64]				Δ mean titers (MD)	12	4.17	0.37, 7.96	0.03	94
Lei [58]	Probiotics		A/H1N1	SC (OR)	4	1.91	0.68, 5.38	0.22	56
Yeh [64]				Δ mean titers (MD)	7	4.71	0.53, 8.89	0.03	97
Lei [58]			A/H3N2	SC (OR)	4	3.52	1.45, 8.53	0.005	63
Yeh [64]				Δ mean titers (MD)	7	16.86	0.87, 32.85	0.04	100
Yeh [64]			B	Δ mean titers (MD)	7	3.04	-0.77, 6.85	0.12	96
Lei [58]	Prebiotics		A/H1N1	SC (OR)	2	0.99	0.54, 1.83	0.98	0
Yeh [64]				Δ mean titers (MD)	5	35.15	0.31, 70.00	0.05	72
Lei [58]			A/H3N2	SC (OR)	2	1.31	0.22, 7.98	0.77	75
Yeh [64]				Δ mean titers (MD)	5	18.66	-13.24, 50.56	0.25	69
Yeh [64]			B	Δ mean titers (MD)	5	20.68	-9.07, 50.42	0.17	79

CI: confidence interval; ES: effect size; MD: mean difference; OR: odds ratio; SC: seroconversion rate; SP: seroprotection rate

Table S4. Subgroup analysis (by supplement type and age-class) of the summary evidence of the effect of probiotic, prebiotic or symbiotic use on seroconversion rates.

**Parameter**		**A/H1N1**	**A/H3N2**	**B**
	k	3	2	3
	N	71/73	52/49	96/91
**Probiotics**	**OR RE (95% CI)**	*2.89 (1.19, 6.99)*	2.37 (0.41, 13.78)	1.81 (0.90, 3.65)
	**OR FE (95% CI)**	*2.89 (1.19, 6.99)*	1.59 (0.71, 3.58)	1.81 (0.90, 3.65)
	I^2, %	0	50.2	0
	k	4	3	3
	N	132/125	109/104	96/83
**Prebiotics**	**OR RE (95% CI)**	2.21 (0.78, 6.21)	1.42 (0.42, 4.77)	1.16 (0.58, 2.36)
	**OR FE (95% CI)**	1.58 (0.93, 2.71)	0.95 (0.52, 1.75)	1.16 (0.58, 2.36)
	I^2, %	56.8	48.1	0
	k	3	3	3
	N	74/76	74/76	74/76
**Symbiotics**	**OR RE (95% CI)**	0.67 (0.24, 1.85)	1.14 (0.33, 3.92)	0.58 (0.24, 1.41)
	**OR FE (95% CI)**	0.73 (0.36, 1.50)	1.06 (0.53, 2.10)	0.60 (0.27, 1.34)
	I^2, %	45.9	68.9	14.3
	k	4	3	3
	N	89/94	70/70	70/70
**Adults**	**OR RE (95% CI)**	1.28 (0.61, 2.72)	*2.32 (1.07, 5.03)*	0.84 (0.39, 1.79)
	**OR FE (95% CI)**	1.28 (0.61, 2.72)	*2.32 (1.07, 5.03)*	0.84 (0.39, 1.79)
	I^2, %	0	0	0
	k	6	5	6
	N	188/180	165/159	196/180
**Elderly**	**OR RE (95% CI)**	1.86 (0.77, 4.47)	0.92 (0.48, 1.77)	1.31 (0.79, 2.18)
	**OR FE (95% CI)**	1.47 (0.94, 2.32)	0.86 (0.55, 1.37)	1.31 (0.79, 2.18)
	I^2, %	65.1	35.6	0

Statistically significant estimates are evidenced *in italics*; CI: confidence interval; FE: fixed-effects model; OR: odds ratio; RE: random-effects model

Table S5. Subgroup analysis (by supplement type and age-class) of the summary evidence of the effect of probiotic, prebiotic or symbiotic use on seroprotection rates.

**Parameter**		**A/H1N1**	**A/H3N2**	**B**
**Probiotics**	***k***	5	4	4
	N	630/644	611/622	611/620
	**OR RE (95% CI)**	1.76 (0.92, 3.37)	1.20 (0.49, 2.94)	1.02 (0.75, 1.40)
	**OR FE (95% CI)**	1.15 (0.86, 1.53)	1.37 (0.71, 2.66)	1.02 (0.75, 1.40)
	**I^2, %**	51.8	34.8	0
**Prebiotics**	***k***	4	3	3
	N	132/125	109/104	104/103
	**OR RE (95% CI)**	*2.72 (1.14, 6.50)*	*3.86 (1.33, 11.19)*	0.88 (0.48, 1.61)
	**OR FE (95% CI)**	*2.36 (1.36, 4.08)*	*3.86 (1.33, 11.19)*	0.88 (0.48, 1.61)
	**I^2, %**	43.1	0	0
**Symbiotics**	***k***	4	4	4
	N	83/86	85/86	85/87
	**OR RE (95% CI)**	0.69 (0.24, 2.00)	2.38 (0.60, 9.44)	0.60 (0.21, 1.74)
	**OR FE (95% CI)**	0.64 (0.28, 1.43)	2.27 (0.91, 5.65)	0.66 (0.31, 1.42)
	**I^2, %**	32.5	54.5	37.6
**Adults**	***k***	5	4	4
	N	620/636	601/612	601/612
	**OR RE (95% CI)**	0.92 (0.68, 1.25)	1.97 (0.79, 4.91)	0.96 (0.68, 1.34)
	**OR FE (95% CI)**	0.92 (0.68, 1.25)	1.85 (0.87, 3.94)	0.96 (0.68, 1.34)
	**I^2, %**	0	29.0	46.4
**Elderly**	***k***	8	7	7
	N	225/219	204/200	199/198
	**OR RE (95% CI)**	*2.40 (1.25, 4.60)*	1.87 (0.78, 4.49)	0.92 (0.61, 1.40)
	**OR FE (95% CI)**	*2.17 (1.45, 3.26)*	*2.00 (1.08, 3.71)*	0.92 (0.61, 1.40)
	**I^2, %**	52.7	38.8	0

Statistically significant estimates are evidenced *in italics*; CI: confidence interval; FE: fixed-effects model; OR: odds ratio; RE: random-effects model

Table S6. Subgroup analysis (by supplement type and age-class) of the summary evidence of the effect of probiotic, prebiotic or symbiotic use on post-vaccination hemagglutination-inhibition titers.

**Parameter**		**A/H1N1**	**A/H3N2**	**B**
**Probiotics**	***k***	6	5	5
	N	233/232	214/208	214/208
	***g* RE (95% CI)**	0.08 (-0.10, 0.27)	0.06 (-0.13, 0.26)	0.00 (-0.19, 0.19)
	***g* FE (95% CI)**	0.08 (-0.10, 0.27)	0.06 (-0.13, 0.26)	0.00 (-0.19, 0.19)
	**I^2, %**	0	0	0
**Prebiotics**	***k***	2	2	2
	N	92/90	92/90	92/90
	***g* RE (95% CI)**	0.07 (-0.23, 0.36)	0.03 (-0.26, 0.32)	0.04 (-0.25, 0.33)
	***g* FE (95% CI)**	0.07 (-0.23, 0.36)	0.03 (-0.26, 0.32)	0.04 (-0.25, 0.33)
	**I^2, %**	0	0	0
**Symbiotics**	***k***	3	3	3
	N	74/76	74/76	74/76
	***g* RE (95% CI)**	-0.08 (-0.40, 0.24)	0.01 (-0.31, 0.33)	-0.06 (-0.38, 0.26)
	***g* FE (95% CI)**	-0.08 (-0.40, 0.24)	0.01 (-0.31, 0.33)	-0.06 (-0.38, 0.26)
	**I^2, %**	0	0	0
**Adults**	***k***	3	2	2
	N	67/73	48/49	48/49
	***g* RE (95% CI)**	0.15 (-0.19, 0.49)	0.08 (-0.31, 0.48)	-0.02 (-0.42, 0.37)
	***g* FE (95% CI)**	0.15 (-0.19, 0.48)	0.08 (-0.31, 0.48)	-0.02 (-0.42, 0.37)
	**I^2, %**	3.6	0	0
**Elderly**	***k***	8	8	8
	N	332/325	332/325	332/325
	***g* RE (95% CI)**	0.03 (-0.12, 0.18)	0.04 (-0.11, 0.19)	0.00 (-0.15, 0.15)
	***g* FE (95% CI)**	0.03 (-0.12, 0.18)	0.08 (-0.11, 0.19)	0.00 (-0.15, 0.15)
	**I^2, %**	0	0	0

CI: confidence interval; FE: fixed-effects model; RE: random-effects model

Table S7. Pooled estimates extracted from the available meta-analyses [62] of the effect of vitamin D deficiency on the influenza vaccine-induced immune response (all models are random-effects).

**Parameter**		**A/H1N1**	**A/H3N2**	**B**
**Seroconversion rate**	***k***	6	4	4
	N	383/1178	358/1079	358/1079
	**OR (95% CI)**	1.10 (0.79, 1.54)	0.98 (0.76, 1.25)	0.98 (0.75, 1.27)
	**I^2, %**	25	0	0
**Seroprotection rate**	***k***	6	4	4
	N	373/1144	358/1079	358/1019
	**OR (95% CI)**	1.00 (0.52, 1.92)	*0.63 (0.43, 0.91)*	*0.68 (0.50, 0.93)*
	**I^2, %**	75	36	1

Statistically significant estimates are reported *in italics*; CI: confidence interval; OR: odds ratio

Table S8. Pooled estimates extracted from the available meta-analyses of the effect of immunosuppressive conditions on the influenza vaccine-induced immune response.

**Study**	**Condition**	**Population**	**Virus**	**Parameter (ES)**	***k***	**Model**	**ES**	**95% CI**	***P***	***I*²****, %**
Beck [42]	Any immuno-suppression	Any	A/H1N1	SC (OR)	50	RE	0.55	0.43, 0.71	<0.001	53.2
			A/H3N2		47	RE	0.55	0.41, 0.73	<0.001	66.9
			B		44	RE	0.48	0.36, 0.62	<0.001	54.3
			A/H1N1	SP (OR)	37	RE	0.36	0.26, 0.51	<0.001	56.9
			A/H3N2		35	RE	0.39	0.26, 0.59	<0.001	64.1
			B		37	RE	0.37	0.25, 0.53	<0.001	65.1
Beck [44]	HIV		A/H1N1	SC (OR)	17	FE	0.51	0.40, 0.66	<0.001	19.5
			A/H3N2		15	RE	0.47	0.27, 0.79	0.005	69.4
			B		15	FE	0.34	0.26, 044	<0.001	36.7
			A/H1N1	SP (OR)	7	RE	0.28	0.12, 0.65	0.003	69.8
			A/H3N2		7	RE	0.28	0.09, 0.80	0.02	69.7
			B		7	RE	0.24	0.09, 0.60	0.002	64.3
	Cancer		A/H1N1	SC (OR)	12	FE	0.31	0.22, 0.43	<0.001	34.8
			A/H3N2		12	RE	0.39	0.21, 0.71	<0.001	66.6
			B		8	RE	0.37	0.20, 0.68	0.001	46.5
			A/H1N1	SP (OR)	10	RE	0.30	0.15, 0.61	0.001	64.3
			A/H3N2		10	RE	0.30	0.14, 0.63	0.002	62.6
			B		9	RE	0.30	0.14, 0.67	0.003	68.4
	Transplantation		A/H1N1	SC (OR)	10	RE	0.76	0.38, 1.51	>0.05	57.8
			A/H3N2		10	RE	0.38	0.23, 0.62	<0.001	42.8
			B		10	RE	0.48	0.27, 0.86	0.01	55.2
			A/H1N1	SP (OR)	10	FE	0.28	0.16, 0.47	<0.001	7.3
			A/H3N2		7	RE	0.29	0.09, 0.92	0.04	78.4
			B		9	RE	0.36	0.19, 0.70	0.002	59.0
	Autoimmune diseases treated with ISs		A/H1N1	SC (OR)	8	RE	0.90	0.45, 1.80	>0.05	68.8
			A/H3N2		7	FE	1.54	1.03, 2.32	0.04	32.1
			B		7	RE	0.98	0.43, 2.24	>0.05	65.1
			A/H1N1	SP (OR)	7	FE	0.49	0.29, 0.84	0.01	0
			A/H3N2		9	FE	0.71	0.43, 1.17	>0.05	3.8
			B		9	RE	0.48	0.22, 1.05	>0.05	65.9
	Respiratory diseases on ISs		A/H1N1	SC (OR)	2	RE	0.96	0.33, 2.79	>0.05	46.3
			A/H3N2		2	RE	0.93	0.30, 2.85	>0.05	53.3
			B		2	FE	0.29	0.14, 0.59	0.001	23.6
Eckerle [45]	Solid organ transplantation (any)	Any	A/H1N1	Response rate (RD)[†]	19	FE	-0.12	-0.16, -0.08	<0.05	88.0
					19	RE	-0.16	-0.25, -0.06	<0.05	
			A/H3N2		18	FE	-0.15	-0.19, -0.11	<0.05	79.2
					18	RE	-0.15	-0.23, -0.07	<0.05	
			B		19	FE	-0.12	-0.16, -0.07	<0.05	83.7
					19	RE	-0.10	-0.19, -0.01	<0.05	
	Renal transplantation		A/H1N1		9	FE	-0.12	-0.19, -0.06	<0.05	87.3
					9	RE	-0.15	-0.32, 0.02	>0.05	
			A/H3N2		9	FE	-0.15	-0.22, -0.09	<0.05	53.7
					9	RE	-0.13	-0.22, -0.04	<0.05	
			B		9	FE	0.03	-0.03, 0.09	>0.05	57.1
					9	RE	0.00	-0.09, 0.09	>0.05	
	Liver transplantation		A/H1N1		5	FE	-0.07	-0.14, 0.01	>0.05	0
					5	RE	-0.07	-0.13, -0.01	<0.05	
			A/H3N2		5	FE	-0.06	-0.13, 0.00	0.05	83.1
					5	RE	-0.10	-0.25, 0.06	>0.05	
			B		5	FE	-0.03	-0.10, 0.04	>0.05	13.7
					5	RE	-0.04	-0.12, 0.03	>0.05	
	Heart transplantation		A/H1N1		3	FE	-0.06	-0.14, -0.01	<0.05	94.9
					3	RE	-0.19	-0.75, 0.37	>0.05	
			A/H3N2		2	FE	-0.27	-0.37, -0.17	<0.05	0
					2	RE	-0.27	-0.37, -0.17	<0.05	
			B		3	FE	-0.46	-0.57, -0.35	<0.05	48.1
					3	RE	-0.41	-0.59, -0.23	<0.05	

Hua [47]	RA patients on methotrexate	Adults	A/H1N1	ARR (OR)	2	FE	1.36	0.69, 2.68	0.37	0
			A/H3N2		2	FE	1.33	0.70, 2.53	0.39	0
			B		2	FE	1.28	0.64, 2.56	0.48	0
	RA patients on rituximab		A/H1N1		2	FE	0.44	0.17, 1.12	0.08	56
			A/H3N2		2	FE	0.11	0.04, 0.31	<0.0001	0
			B		2	FE	0.29	0.10, 0.81	0.02	37
	RA patients on αTNF		A/H1N1		4	RE	0.93	0.36, 2.37	0.88	74
			A/H3N2		4	RE	0.79	0.34, 1.83	0.59	68
			B		4	RE	0.79	0.37, 1.70	0.55	57
Huang [54]	SLE	Any	A/H1N1	SC (RR)	13	RE	0.71	0.62, 0.81	<0.00001	56
			A/H3N2		7	RE	0.73	0.53, 1.01	0.06	72
			B		4	RE	0.66	0.52, 0.82	0.0003	0
			A/H1N1	SP (RR)	12	RE	0.79	0.73, 0.87	<0.00001	46
			A/H3N2		6	RE	0.84	0.68, 1.03	0.09	68
			B		5	RE	0.75	0.65, 0.87	0.0002	0
Liao [55]			A/H1N1	SC (OR)	15	RE	0.39	0.27, 0.57	<0.00001	65
			A/H3N2		6	RE	0.62	0.21, 1.79	0.37	77
			B		6	FE	0.47	0.29, 0.76	0.002	0
			A/H1N1	SP (OR)	18	RE	0.36	0.27, 0.50	<0.00001	35
			A/H3N2		8	RE	0.48	0.24, 0.93	0.03	33
			B		6	RE	0.55	0.24, 1.25	0.16	42
Pugès [56]		Adults	A/H1N1	SC (OR)	12	RE	0.38	0.27, 0.54	<0.00001	39
			A/H3N2		11	RE	0.66	0.36, 1.23	0.19	61
			B		5	RE	0.51	0.20, 1.28	0.15	66
			A/H1N1	SP (OR)	11	RE	0.36	0.28, 0.47	<0.00001	25
			A/H3N2		6	FE	0.26	0.14, 0.50	<0.0001	21
			B		5	RE	0.93	0.42, 2.08	0.86	56
Huang [57]	RA		A/H1N1	SC (RR)	12	RE	0.78	0.68, 0.90	0.0004	43
			A/H3N2		8	RE	1.11	0.93, 1.32	0.24	14
			B		9	RE	0.84	0.62, 1.14	0.27	47
			A/H1N1	SP (RR)	9	RE	0.72	0.60, 0.86	0.0005	89
			A/H3N2		5	RE	0.96	0.82, 1.13	0.64	26
			B		5	RE	0.95	0.84, 1.08	0.44	28
Subesinghe [63]	RA patients on methotrexate		A/H1N1	SP (RR)	5	RE	0.88	0.69, 1.11	0.27	81
			A/H3N2		2	RE	0.94	0.85, 1.04	0.22	0
			B		2	RE	1.15	0.63, 2.10	0.64	92
	RA patients on αTNF		A/H1N1	SP (RR)	7	RE	0.86	0.72, 1.04	0.12	70
			A/H3N2		4	RE	0.98	0.74, 1.31	0.89	65
			B		4	RE	1.38	0.70, 2.72	0.35	91

†Seroconversion and seroprotection rates were probably pooled together; ARR: absolute risk reduction; CI: confidence interval; ES: effect size; FE: fixed-effects model; HIV: human immunodeficiency virus; IS: immunosupressant; OR: odds ratio; RCT: randomized controlled trial; RA: rheumatoid arthritis; RE: random-effects model; RR: risk ratio; SC: seroconversion rate; SLE: systemic lupus erythematosus; SP: seroprotection rate; TNF: tumor necrosis factor

Table S9. Sensitivity analysis (on excluding studies with imputed standard deviations) on the effect of any immunosuppressive condition on post-vaccination hemagglutination-inhibition titers.

Parameter	A/H1N1	A/H3N2	B
k	52	35	35
N	5616/2425	2116/1174	2198/1234
***g* RE (95% CI)**	-0.34 (-0.45, -0.23)	-0.53 (-0.68, -0.39)	-0.36 (-0.50, -0.21)
***P* RE**	$9 \cdot 10^{-10}$	$3 \cdot 10^{-13}$	$1 \cdot 10^{-6}$
**95% PI**	-0.98, 0.30	-1.23, 0.16	-1.05, 0.34
***g* FE (95% CI)**	-0.29 (-0.34, -0.24)	-0.51 (-0.58, -0.43)	-0.30 (-0.38, -0.23)
***P* FE**	$<1 \cdot 10^{-15}$	$<1 \cdot 10^{-15}$	$3 \cdot 10^{-15}$
**I^2, %**	73.9	68.6	69.7
**τ^2**	0.10	0.12	0.12
**SSE, *P***	0.11	0.25	0.062
**LS**	Yes	Yes	No
**CES**	II	II	III

CES: cumulative evidence synthesis class; CI: confidence interval; FE: fixed-effects model; LS: the largest study has a statistically significant effect size; PI: prediction interval; RE: random-effects model; SSE: small-study effect test

Table S10. Multivariable meta-regression analysis in order to predict the observed pooled estimates of seroconversion rates.

**Parameter**	**Variable**	**Univariable model**		**Full model**	
		b (SE)	*P*	*b* (SE)	*P*
**Publication in year**	<2000	Ref	–	Ref	–
	≥2000	0.06 (0.13)	0.64	0.03 (0.13)	0.84
**Total sample size**	<100	Ref	–	Ref	–
	≥100	0.14 (0.11)	0.21	0.14 (0.12)	0.25
**Age**	Children	Ref	–	Ref	–
	Adults	-0.02 (0.14)	0.86	-0.11 (0.14)	0.44
**Virus**	A/H1N1	Ref	–	Ref	–
	A/H3N2	0.03 (0.13)	0.82	0.08 (0.13)	0.56
	B	0.07 (0.14)	0.62	0.11 (0.14)	0.42
**Immunosuppression category**	Transplantation	Ref	–	Ref	–
	Cancer	-0.27 (0.17)	0.12	-0.26 (0.18)	0.14
	HIV	-0.05 (0.17)	0.79	-0.07 (0.18)	0.69
	RDs	0.28 (0.15)	0.070	0.27 (0.16)	0.091
	Other/Mixed	-0.11 (0.19)	0.57	-0.17 (0.20)	0.41

HIV: human immunodeficiency virus; RD: rheumatic disease; SE: standard error

Table S11. Multivariable meta-regression analysis in order to predict the observed pooled estimates of seroprotection rates.

**Parameter**	**Variable**	**Univariable model**		**Full model**	
		b (SE)	*P*	*b* (SE)	*P*
**Publication in year**	<2000	Ref	–	Ref	–
	≥2000	0.32 (0.18)	0.076	0.13 (0.20)	0.50
**Total sample size**	<100	Ref	–	Ref	–
	≥100	-0.02 (0.14)	0.88	0.03 (0.15)	0.85
**Age**	Children	Ref	–	Ref	–
	Adults	-0.17 (0.18)	0.35	-0.18 (0.18)	0.32
**Virus**	A/H1N1	Ref	–	Ref	–
	A/H3N2	-0.19 (0.17)	0.26	-0.14 (0.17)	0.39
	B	0.23 (0.16)	0.16	0.26 (0.16)	0.11
**Immunosuppression category**	Transplantation	Ref	–	Ref	–
	Cancer	0.27 (0.23)	0.23	0.24 (0.24)	0.31
	HIV	-0.11 (0.25)	0.66	-0.13 (0.25)	0.62
	RDs	0.42 (0.19)	0.026	0.39 (0.20)	0.051
	Other/Mixed	0.35 (0.24)	0.14	0.26 (0.26)	0.31

HIV: human immunodeficiency virus; RD: rheumatic disease; SE: standard error

Table S12. Multivariable meta-regression analysis in order to predict the observed pooled estimates of post-vaccination hemagglutination-inhibition titers.

**Parameter**	**Variable**	**Univariable model**		**Full model**	
		b (SE)	*P*	*b* (SE)	*P*
**Publication in year**	<2000	Ref	–	Ref	–
	≥2000	0.16 (0.07)	0.016	0.06 (0.07)	0.38
**Total sample size**	<100	Ref	–	Ref	–
	≥100	0.05 (0.06)	0.42	0.04 (0.06)	0.51
**Age**	Children	Ref	–	Ref	–
	Adults	0.01 (0.07)	0.85	0.00 (0.07)	0.99
**Virus**	A/H1N1	Ref	–	Ref	–
	A/H3N2	-0.08 (0.07)	0.80	-0.05 (0.06)	0.39
	B	0.02 (0.07)	0.80	0.05 (0.07)	0.45
**Immunosuppression category**	Transplantation	Ref	–	Ref	–
	Cancer	0.14 (0.10)	0.13	0.14 (0.10)	0.18
	HIV	0.01 (0.10)	0.89	0.01 (0.10)	0.92
	RDs	0.41 (0.08)	<0.0001	0.40 (0.09)	<0.0001
	Other/Mixed	0.22 (0.10)	0.32	0.20 (0.10)	0.060

HIV: human immunodeficiency virus; RD: rheumatic disease; SE: standard error

Table S13. Summary evidence of the effect of rheumatic diseases on the influenza vaccine-induced immune response, by immunogenicity parameter and viral (sub)type.

Parameter	A/H1N1	A/H3N2	B
**Seroconversion rate**			
k	38	26	21
N	4676/1825	1245/657	1091/568
**OR RE (95% CI)**	0.57 (0.43, 0.76)	0.78 (0.56, 1.10)	0.76 (0.54, 1.06)
***P* RE**	0.0001	0.16	0.11
**95% PI**	0.15, 2.22	0.22, 2.81	0.26, 2.18
**OR FE (95% CI)**	0.57 (0.49, 0.65)	0.83 (0.67, 1.03)	0.75 (0.59, 0.95)
***P* FE**	$<1 \cdot 10^{-15}$	0.090	0.016
**I^2, %**	69.8	54.6	45.5
**τ^2**	0.46	0.39	0.26
**SSE, *P***	0.75	0.83	0.46
**LS**	Yes	No	Yes
**CES**	III	ns	IV
**Seroprotection rate**			
k	42	28	27
N	5108/1971	1618/770	1563/707
**OR RE (95% CI)**	0.47 (0.36, 0.60)	0.41 (0.28, 0.61)	0.76 (0.47, 1.25)
***P* RE**	$3 \cdot 10^{-9}$	0.00001	0.28
**95% PI**	0.19, 1.16	0.13, 1.31	0.11, 5.52
**OR FE (95% CI)**	0.47 (0.40, 0.56)	0.40 (0.30, 0.55)	0.84 (0.64, 1.11)
***P* FE**	$<1 \cdot 10^{-15}$	$8 \cdot 10^{-9}$	0.22
**I^2, %**	38.7	30.3	64.1
**τ^2**	0.20	0.31	0.96
**SSE, *P***	0.96	0.17	0.31
**LS**	Yes	Yes	Yes
**CES**	II	II	ns
**Post-vaccination hemagglutination-inhibition titer**			
k	38	27	22
N	4479/1810	1591/833	1415/668
***g* RE (95% CI)**	-0.21 (-0.30, -0.11)	-0.26 (-0.39, -0.12)	-0.05 (-0.21, 0.10)
***P* RE**	0.00002	0.0003	0.50
**95% PI**	-0.63, 0.21	-0.80, 0.29	-0.61, 0.51
***g* FE (95% CI)**	-0.23 (-0.29, -0.17)	-0.28 (-0.37, -0.19)	-0.05 (-0.15, 0.05)
***P* FE**	$6 \cdot 10^{-15}$	$6 \cdot 10^{-10}$	0.31
**I^2, %**	55.5	57.0	58.5
**τ^2**	0.04	0.07	0.08
**SSE, *P***	0.29	0.26	0.85
**LS**	Yes	No	Yes
**CES**	III	III	ns

CES: cumulative evidence synthesis class; CI: confidence interval; FE: fixed-effects model; LS: the largest study has a statistically significant effect size; OR: odds ratio; PI: prediction interval; RE: random-effects model; SSE: small-study effect test

Table S14. Summary evidence of the effect of rheumatoid arthritis on the influenza vaccine-induced immune response, by immunogenicity parameter and viral (sub)type.

**Parameter**	**A/H1N1**	**A/H3N2**	**B**
**Seroconversion rate**			
k	16	12	11
N	1030/719	517/303	502/273
**OR RE (95% CI)**	0.64 (0.45, 0.91)	0.93 (0.62, 1.40)	0.75 (0.45, 1.26)
***P* RE**	0.014	0.73	0.27
**95% PI**	0.23, 1.80	0.39, 2.25	0.22, 2.55
**OR FE (95% CI)**	0.55 (0.45, 0.68)	0.97 (0.71, 1.32)	0.72 (0.51, 1.01)
***P* FE**	$8 \cdot 10^{-8}$	0.84	0.060
**I^2, %**	53.8	33.7	47.0
**τ^2**	0.24	0.16	0.32
**SSE, *P***	0.13	0.49	0.63
**LS**	Yes	No	Yes
**CES**	IV	ns	ns
**Seroprotection rate**			
k	19	15	15
N	1330/827	816/412	815/403
**OR RE (95% CI)**	0.37 (0.23, 0.53)	0.36 (0.20, 0.63)	0.84 (0.40, 1.79)
***P* RE**	0.00001	0.0004	0.65
**95% PI**	0.11, 1.26	0.09, 1.48	0.08, 8.96
**OR FE (95% CI)**	0.39 (0.20, 0.51)	0.37 (0.25, 0.55)	0.95 (0.64, 1.41)
***P* FE**	$9 \cdot 10^{-13}$	$9.96 \cdot 10^{-7}$	0.81
**I^2, %**	47.0	39.8	67.7
**τ^2**	0.35	0.44	1.31
**SSE, *P***	0.74	0.60	0.53
**LS**	Yes	Yes	Yes
**CES**	II	IV	ns
**Post-vaccination hemagglutination-inhibition titer**			
k	15	13	12
N	1283/832	769/417	753/378
***g* RE (95% CI)**	-0.34 (-0.47, -0.21)	-0.27 (-0.42, -0.11)	-0.11 (-0.32, 0.10)
***P* RE**	$3 \cdot 10^{-7}$	0.0009	0.31
**95% PI**	-0.37, -0.04	-0.60, 0.07	-0.69, 0.47
***g* FE (95% CI)**	-0.37 (-0.47, -0.27)	-0.26 (-0.38, -0.13)	-0.10 (-0.23, 0.03)
***P* FE**	$1 \cdot 10^{-13}$	0.00008	0.14
**I^2, %**	30.8	29.3	58.1
**τ^2**	0.02	0.02	0.08
**SSE, *P***	0.22	0.62	0.71
**LS**	Yes	No	No
**CES**	I	IV	ns

CES: cumulative evidence synthesis class; CI: confidence interval; FE: fixed-effects model; LS: the largest study has a statistically significant effect size; OR: odds ratio; PI: prediction interval; RE: random-effects model; SSE: small-study effect test

Table S15. Summary evidence of the effect of systemic lupus erythematosus on the influenza vaccine-induced immune response, by immunogenicity parameter and viral (sub)type.

Parameter	A/H1N1	A/H3N2	B
**Seroconversion rate**			
k	17	10	5
N	1767/924	344/251	231/129
**OR RE (95% CI)**	0.35 (0.23, 0.53)	0.55 (0.27, 1.12)	0.57 (0.35, 0.93)
***P* RE**	$9{\cdot}10^{-7}$	0.10	0.025
**95% PI**	0.10, 1.29	0.08, 3.67	0.35, 0.93
**OR FE (95% CI)**	0.38 (0.31, 0.46)	0.50 (0.34, 0.75)	0.57 (0.35, 0.93)
***P* FE**	$<1{\cdot}10^{-15}$	0.0008	0.025
**I^2, %**	68.8	65.2	0
**τ^2**	0.40	0.80	0
**SSE, *P***	0.79	0.14	NA
**LS**	Yes	Yes	No
**CES**	II	ns	IV
**Seroprotection rate**			
k	16	7	6
N	1784/945	303/201	249/147
**OR RE (95% CI)**	0.38 (0.27, 0.54)	0.40 (0.21, 0.76)	0.52 (0.25, 1.09)
***P* RE**	$2{\cdot}10^{-8}$	0.0054	0.083
**95% PI**	0.17, 0.85	0.14, 1.12	0.12, 2.21
**OR FE (95% CI)**	0.38 (0.30, 0.47)	0.39 (0.23, 0.68)	0.60 (0.36, 0.99)
***P* FE**	$<1{\cdot}10^{-15}$	0.0008	0.045
**I^2, %**	36.3	22.8	49.3
**τ^2**	0.14	0.17	0.40
**SSE, *P***	0.75	NA	NA
**LS**	Yes	Yes	Yes
**CES**	I	IV	IV
**Post-vaccination hemagglutination-inhibition titer**			
k	13	9	NA
N	1181/758	323/258	NA
***g* RE (95% CI)**	-0.42 (-0.59, 0.25)	-0.23 (-0.48, 0.03)	NA
***P* RE**	$1{\cdot}10^{-6}$	0.082	NA
**95% PI**	-0.88, 0.04	-0.82, 0.37	NA
***g* FE (95% CI)**	-0.47 (-0.56, -0.37)	-0.26 (-0.43, -0.09)	NA
***P* FE**	$<1{\cdot}10^{-15}$	0.0026	NA
**I^2, %**	56.7	52.0	NA
**τ^2**	0.05	0.08	NA
**SSE, *P***	0.87	NA	NA
**LS**	Yes	Yes	NA
**CES**	III	ns	NA

CES: cumulative evidence synthesis class; CI: confidence interval; FE: fixed-effects model; LS: the largest study has a statistically significant effect size; OR: odds ratio; PI: prediction interval; RE: random-effects model; SSE: small-study effect test

Table S16. Summary evidence of the effect of inflammatory bowel disease on the influenza vaccine-induced immune response, by immunogenicity parameter and viral (sub)type.

Parameter	A/H1N1	A/H3N2	B
**Seroconversion rate**			
k	2	NA	NA
N	154/65	NA	NA
**OR RE (95% CI)**	0.54 (0.15, 1.96)	NA	NA
***P* RE**	0.35	NA	NA
**95% PI**	0.07, 4.08	NA	NA
**OR FE (95% CI)**	0.50 (0.26, 0.98)	NA	NA
***P* FE**	0.043	NA	NA
**I^2, %**	73.0	NA	NA
**τ^2**	0.63	NA	NA
**SSE, *P***	NA	NA	NA
**LS**	Yes	NA	NA
**CES**	ns	NA	NA
**Seroprotection rate**			
k	5	3	3
N	309/104	108/45	108/45
**OR RE (95% CI)**	0.80 (0.35, 1.86)	0.74 (0.23, 2.39)	1.12 (0.07, 17.76)
***P* RE**	0.61	0.61	0.94
**95% PI**	0.18, 3.46	0.23, 2.39	0.01, 120.4
**OR FE (95% CI)**	0.80 (0.45, 1.41)	0.74 (0.23, 2.39)	0.28 (0.10, 0.78)
***P* FE**	0.44	0.61	0.015
**I^2, %**	43.7	0	61.5
**τ^2**	0.37	0	3.7
**SSE, *P***	NA	NA	NA
**LS**	No	No	Yes
**CES**	ns	ns	ns
**Post-vaccination hemagglutination-inhibition titer**			
k	2	NA	NA
N	201/59	NA	NA
***g* RE (95% CI)**	-0.30 (-0.59, 0.00)	NA	NA
***P* RE**	0.043	NA	NA
**95% PI**	-0.59, 0.00	NA	NA
***g* FE (95% CI)**	-0.30 (-0.59, 0.00)	NA	NA
***P* FE**	0.043	NA	NA
**I^2, %**	0	NA	NA
**τ^2**	0	NA	NA
**SSE, *P***	NA	NA	NA
**LS**	No	NA	NA
**CES**	IV	NA	NA

CES: cumulative evidence synthesis class; CI: confidence interval; FE: fixed-effects model; LS: the largest study has a statistically significant effect size; OR: odds ratio; PI: prediction interval; RE: random-effects model; SSE: small-study effect test

Table S17. Summary evidence of the effect of human immunodeficiency virus (HIV) infection on the influenza vaccine-induced immune response, by immunogenicity parameter and viral (sub)type.

Parameter	A/H1N1	A/H3N2	B
**Seroconversion rate**			
k	20	17	15
N	828/1059	594/803	558/742
**OR RE (95% CI)**	0.51 (0.35, 0.74)	0.46 (0.26, 0.82)	0.40 (0.29, 0.57)
***P* RE**	0.0005	0.0082	$2 \cdot 10^{-7}$
**95% PI**	0.14, 1.81	0.06, 3.41	0.18, 0.88
**OR FE (95% CI)**	0.54 (0.43, 0.67)	0.50 (0.38, 0.64)	0.37 (0.29, 0.49)
***P* FE**	$4 \cdot 10^{-8}$	$1 \cdot 10^{-7}$	$2 \cdot 10^{-13}$
**I^2, %**	59.4	75.2	30.4
**τ^2**	0.38	0.95	0.13
**SSE, *P***	0.47	0.93	0.22
**LS**	Yes	Yes	Yes
**CES**	IV	IV	IV
**Seroprotection rate**			
k	14	11	9
N	601/936	367/680	331/619
**OR RE (95% CI)**	0.35 (0.19, 0.64)	0.21 (0.13, 0.34)	0.33 (0.15, 0.72)
***P* RE**	0.0006	$2 \cdot 10^{-10}$	0.0059
**95% PI**	0.06, 1.91	0.13, 0.34	0.05, 2.12
**OR FE (95% CI)**	0.29 (0.21, 0.39)	0.21 (0.13, 0.34)	0.25 (0.16, 0.39)
***P* FE**	$<1 \cdot 10^{-15}$	$2 \cdot 10^{-10}$	$6 \cdot 10^{-10}$
**I^2, %**	64.5	0	59.3
**τ^2**	0.65	0	0.74
**SSE, *P***	0.70	0.60	NA
**LS**	Yes	Yes	Yes
**CES**	IV	IV	IV
**Post-vaccination hemagglutination-inhibition titer**			
k	17	15	12
N	788/1100	554/844	518/783
***g* RE (95% CI)**	-0.51 (-0.75, -0.27)	-0.77 (-1.11, -0.42)	-0.54 (-0.76, -0.32)
***P* RE**	0.00003	0.00001	$2 \cdot 10^{-6}$
**95% PI**	-1.38, 0.36	-2.03, 0.49	-1.13, 0.05
***g* FE (95% CI)**	-0.43 (-0.53, -0.32)	-0.67 (-0.79, -0.55)	-0.51 (-0.64, -0.38)
***P* FE**	$<1 \cdot 10^{-15}$	$<1 \cdot 10^{-15}$	$2 \cdot 10^{-14}$
**I^2, %**	78.9	86.5	57.9
**τ^2**	0.18	0.38	0.08
**SSE, *P***	0.26	0.43	0.48
**LS**	Yes	Yes	Yes
**CES**	IV	IV	IV

CES: cumulative evidence synthesis class; CI: confidence interval; FE: fixed-effects model; LS: the largest study has a statistically significant effect size; OR: odds ratio; PI: prediction interval; RE: random-effects model; SSE: small-study effect test

Table S18. Summary evidence of the effect of transplantation on the influenza vaccine-induced immune response, by immunogenicity parameter and viral (sub)type.

Parameter	A/H1N1	A/H3N2	B
**Seroconversion rate**			
k	31	26	25
N	1326/1008	955/888	910/820
**OR RE (95% CI)**	0.49 (0.32, 0.75)	0.35 (0.23, 0.54)	0.54 (0.36, 0.82)
***P* RE**	0.0010	$1{\cdot}10^{-6}$	0.0040
**95% PI**	0.07, 3.58	0.07, 1.87	0.11, 2.71
**OR FE (95% CI)**	0.53 (0.43, 0.65)	0.41 (0.33, 0.51)	0.59 (0.47, 0.73)
***P* FE**	$1{\cdot}10^{-9}$	$<1{\cdot}10^{-15}$	$3{\cdot}10^{-6}$
**I^2, %**	74.0	67.3	65.5
**τ^2**	0.99	0.68	0.63
**SSE, *P***	0.80	0.37	0.56
**LS**	Yes	Yes	No
**CES**	IV	IV	IV
**Seroprotection rate**			
k	23	16	17
N	1110/803	693/607	709/606
**OR RE (95% CI)**	0.28 (0.19, 0.42)	0.26 (0.14, 0.47)	0.46 (0.29, 0.74)
***P* RE**	$6{\cdot}10^{-10}$	$8{\cdot}10^{-6}$	0.0013
**95% PI**	0.08, 0.95	0.05, 1.45	0.12, 1.73
**OR FE (95% CI)**	0.28 (0.21, 0.37)	0.30 (0.21, 0.43)	0.40 (0.30, 0.54)
***P* FE**	$<1{\cdot}10^{-15}$	$2{\cdot}10^{-10}$	$4{\cdot}10^{-9}$
**I^2, %**	40.6	53.9	47.9
**τ^2**	0.32	0.69	0.40
**SSE, *P***	0.11	>0.99	0.12
**LS**	Yes	Yes	Yes
**CES**	I	IV	IV
**Post-vaccination hemagglutination-inhibition titer**			
k	17	12	12
N	1063/780	642/607	616/652
***g* RE (95% CI)**	-0.61 (-0.86, -0.35)	-0.89 (-1.15, -0.64)	-0.52 (-0.69, -0.36)
***P* RE**	$3{\cdot}10^{-6}$	$4{\cdot}10^{-12}$	$4{\cdot}10^{-10}$
**95% PI**	-1.57, 0.35	-1.64, -0.14	-0.88, -0.16
***g* FE (95% CI)**	-0.50 (-0.61, -0.40)	-0.81 (-0.93, -0.69)	-0.49 (-0.61, -0.37)
***P* FE**	$<1{\cdot}10^{-15}$	$<1{\cdot}10^{-15}$	$3{\cdot}10^{-15}$
**I^2, %**	82.3	72.8	35.5
**τ^2**	0.22	0.13	0.03
**SSE, *P***	0.12	0.15	0.27
**LS**	No	Yes	No
**CES**	III	IV	IV

CES: cumulative evidence synthesis class; CI: confidence interval; FE: fixed-effects model; LS: the largest study has a statistically significant effect size; OR: odds ratio; PI: prediction interval; RE: random-effects model; SSE: small-study effect test

Table S19. Summary evidence of the effect of cancer on the influenza vaccine-induced immune response, by immunogenicity parameter and viral (sub)type.

Parameter	A/H1N1	A/H3N2	B
**Seroconversion rate**			
k	31	21	17
N	1326/1008	910/577	663/480
**OR RE (95% CI)**	0.49 (0.32, 0.75)	0.44 (0.29, 0.66)	0.41 (0.30, 0.57)
***P* RE**	0.0010	0.00007	$6{\cdot}10^{-8}$
**95% PI**	0.07, 3.58	0.10, 1.86	0.26, 0.67
**OR FE (95% CI)**	0.53 (0.43, 0.65)	0.50 (0.39, 0.64)	0.41 (0.31, 0.56)
***P* FE**	$1{\cdot}10^{-9}$	$4{\cdot}10^{-8}$	$1{\cdot}10^{-8}$
**I^2, %**	74.0	59.6	7.5
**τ^2**	0.99	0.51	0.03
**SSE, *P***	0.80	0.48	0.19
**LS**	Yes	Yes	Yes
**CES**	III	IV	IV
**Seroprotection rate**			
k	23	14	
N	1110/803	565/375	565/375
**OR RE (95% CI)**	0.28 (0.19, 0.42)	0.37 (0.19, 0.73)	0.46 (0.26, 0.79)
***P* RE**	$6{\cdot}10^{-10}$	0.0038	0.0052
**95% PI**	0.08, 0.95	0.05, 2.84	0.10, 2.08
**OR FE (95% CI)**	0.28 (0.21, 0.37)	0.52 (0.37, 0.75)	0.52 (0.36, 0.74)
***P* FE**	$<1{\cdot}10^{-15}$	0.00042	0.00024
**I^2, %**	40.6	65.8	52.7
**τ^2**	0.34	0.95	0.52
**SSE, *P***	0.11	0.42	0.83
**LS**	Yes	Yes	Yes
**CES**	I	IV	IV
**Post-vaccination hemagglutination-inhibition titer**			
k	17	16	13
N	1063/780	565/506	480/401
***g* RE (95% CI)**	-0.61 (-0.86, -0.35)	-0.54 (-0.77, -0.31)	-0.54 (-0.75, -0.36)
***P* RE**	$3{\cdot}10^{-6}$	$6{\cdot}10^{-6}$	$3{\cdot}10^{-7}$
**95% PI**	-1.57, 0.35	-1.33, 0.26	-1.11, 0.03
***g* FE (95% CI)**	-0.50 (-0.61, -0.40)	-0.47 (-0.60, -0.35)	-0.52 (-0.66, -0.38)
***P* FE**	$<1{\cdot}10^{-15}$	$2{\cdot}10^{-13}$	$3{\cdot}10^{-13}$
**I^2, %**	82.3	69.0	52.5
**τ^2**	0.22	0.15	0.07
**SSE, *P***	0.12	0.082	0.49
**LS**	No	No	Yes
**CES**	III	IV	IV

CES: cumulative evidence synthesis class; CI: confidence interval; FE: fixed-effects model; LS: the largest study has a statistically significant effect size; OR: odds ratio; PI: prediction interval; RE: random-effects model; SSE: small-study effect test

Table S20. Subgroup analysis (by age-class) of the summary evidence of the effect of latent cytomegalovirus (CMV) infection on the influenza vaccine-induced immune response.

**Parameter**		**A/H1N1**	**A/H3N2**	**B**
**Seroconversion rate**				
	k	3	NA	NA
	N	85/46	NA	NA
**Adults**	**OR RE (95% CI)**	0.33 (0.04, 2.70)	NA	NA
	**OR FE (95% CI)**	*0.35 (0.12, 0.99)*	NA	NA
	**I^2 , %**	71.5	NA	NA
	k	3	6	2
	N	107/37	662/187	91/27
**Elderly**	**OR RE (95% CI)**	0.63 (0.13, 3.04)	1.04 (0.73, 1.49)	0.56 (0.19, 1.70)
	**OR FE (95% CI)**	0.86 (0.35, 2.11)	1.04 (0.73, 1.49)	0.58 (0.21, 1.55)
	**I^2, %**	63.9	0	18.8
**Seroprotection rate**				
**Adults**	***k***	NA	NA	NA
	N	NA	NA	NA
	**OR RE (95% CI)**	NA	NA	NA
	**OR FE (95% CI)**	NA	NA	NA
	**I^2, %**	NA	NA	NA
**Elderly**	***k***	NA	4	NA
	N	NA	571/160	NA
	**OR RE (95% CI)**	NA	1.08 (0.74, 1.56)	NA
	**OR FE (95% CI)**	NA	1.08 (0.74, 1.56)	NA
	**I^2, %**	NA	0	NA
**Post-vaccination hemagglutination-inhibition titer**				
**Adults**	***k***	5	NA	NA
	N	299/168	NA	NA
	**OR RE (95% CI)**	-0.28 (-0.78, 0.20)	NA	NA
	**OR FE (95% CI)**	-0.12 (-0.32, 0.07)	NA	NA
	**I^2, %**	77.2	NA	NA
**Elderly**	***k***	2	6	NA
	N	72/53	671/229	NA
	**OR RE (95% CI)**	-0.17 (-0.52, 0.19)	-0.08 (-0.26, 0.11)	NA
	**OR FE (95% CI)**	-0.17 (-0.52, 0.19)	-0.07 (-0.22, 0.08)	NA
	**I^2, %**	0	29.9	NA

CI: confidence interval; FE: fixed-effects model; OR: odds ratio; RE: random-effects model

Table S21. Pooled estimates extracted from the available meta-analyses [41] of the effect of psychological stress on the influenza vaccine-induced immune response.

**Stressor**	**Age group**	***k***	***N***	**Model**	**ESR (95% CI)**	***P***
Chronic stress	Any	6	493	FE	-0.18 (-0.28, -0.08)	0.001
Life events	Any	6	514	RE	-0.18 (-0.27, -0.09)	0.0001
Perceived stress	Any	3	151	RE	-0.18 (-0.33, -0.02)	0.02
Any	Younger	6	466	FE	-0.17 (-0.27, -0.07)	0.001
Any	Older	6	568	FE	-0.25 (-0.34, -0.15)	0.0001

CI: confidence interval; ESR: effect size correlation coefficient; FE: fixed-effects model; RE: random-effects model

Table S22. Distribution of cumulative evidence synthesis classes and mean Cohen's *d*s, by viral (sub)type.

Parameter	A/H1N1 (*k*=34)	A/H3N2 (*k*=33)	B (*k*=30)
**CES I, *N* (%)**	4 (12)	0 (0)	0 (0)
**CES II, *N* (%)**	6 (18)	4 (12)	1 (3)
**CES III, *N* (%)**	6 (18)	1 (3)	2 (7)
**CES IV, *N* (%)**	8 (24)	14 (42)	12 (40)
**CES ns, *N* (%)**	10 (29)	14 (42)	15 (50)
**Mean |*d*| (SD)**	0.17 (0.17)	0.16 (0.21)	0.12 (0.16)

CES: cumulative evidence synthesis class; SD: standard deviation
